# Functional analysis of soybean cyst nematode-inducible synthetic promoters and their regulation by biotic and abiotic stimuli in transgenic soybean (*Glycine max*)

**DOI:** 10.3389/fpls.2022.988048

**Published:** 2022-09-09

**Authors:** Mst Shamira Sultana, Mitra Mazarei, Reginald J. Millwood, Wusheng Liu, Tarek Hewezi, C. Neal Stewart

**Affiliations:** ^1^Department of Plant Sciences, University of Tennessee, Knoxville, TN, United States; ^2^Center for Agricultural Synthetic Biology, University of Tennessee, Knoxville, TN, United States; ^3^Department of Horticultural Science, North Carolina State University, Raleigh, NC, United States

**Keywords:** transgenic soybean, synthetic promoters, plant pathogenic nematodes, biotic and abiotic stresses, GUS

## Abstract

We previously identified *cis*-regulatory motifs in the soybean (*Glycine max*) genome during interaction between soybean and soybean cyst nematode (SCN), *Heterodera glycines*. The regulatory motifs were used to develop synthetic promoters, and their inducibility in response to SCN infection was shown in transgenic soybean hairy roots. Here, we studied the functionality of two SCN-inducible synthetic promoters; 4 × M1.1 (TAAAATAAAGTTCTTTAATT) and 4 × M2.3 (ATATAATTAAGT) each fused to the −46 CaMV35S core sequence in transgenic soybean. Histochemical GUS analyses of transgenic soybean plants containing the individual synthetic promoter::GUS construct revealed that under unstressed condition, no GUS activity is present in leaves and roots. While upon nematode infection, the synthetic promoters direct GUS expression to roots predominantly in the nematode feeding structures induced by the SCN and by the root-knot nematode (RKN), *Meloidogyne incognita*. There were no differences in GUS activity in leaves between nematode-infected and non-infected plants. Furthermore, we examined the specificity of the synthetic promoters in response to various biotic (insect: fall armyworm, *Spodoptera frugiperda*; and bacteria: *Pseudomonas syringe* pv. *glycinea*, *P. syringe* pv. *tomato*, and *P. marginalis*) stresses. Additionally, we examined the specificity to various abiotic (dehydration, salt, cold, wounding) as well as to the signal molecules salicylic acid (SA), methyl jasmonate (MeJA), and abscisic acid (ABA) in the transgenic plants. Our wide-range analyses provide insights into the potential applications of synthetic promoter engineering for conditional expression of transgenes leading to transgenic crop development for resistance improvement in plant.

## Introduction

The soybean cyst nematode (SCN; *Heterodera glycines*) is one of the most damaging pathogens in soybean production worldwide. It causes an annual yield loss of 10%–15%; economic loss of more than $1 billion in the United States (Bradley et al., 2021). Soybean cyst nematode is a sedentary endoparasite of roots. Infective juveniles of SCN penetrate host roots and migrate intracellularly within the cortical tissue to the vascular cylinder and then initiate localized reorganization of the host’s cell morphology and physiology, resulting in the formation of specialized feeding sites called syncytia (Hewezi and Baum, 2013, 2017). Soybean cyst nematodes feed exclusively from their syncytia as they develop into adult females and complete their life cycles (Bohlmann, 2015).

Generally, attempted control of this nematode relies on three main approaches, i.e., cultural practices such as crop rotation, chemical application, and resistant cultivars, which are often used in an integrated manner (Sasser and Uzzell, 1991; Concibido et al., 2004; Jones, 2017). Soybean cyst nematode management is complicated by economic restriction to maintain a high proportion of soybean planting in crop rotations, thereby steadily increasing SCN field densities. Although the use of chemical nematicides is effective for SCN control, the residues are highly toxic to the environment and the human body (El-Alfy and Schlenk, 2002). Therefore, the principal management practice for SCN is the use of resistant soybean cultivars. To date, around 90% of the commercial elite soybean cultivars grown carry quantitative trait loci that endow a measure of resistance (Cook et al., 2012). However, the overuse of the same resistant loci leads to the genetic shifts of SCN populations and results in the reduced effectiveness of SCN management practices (Niblack et al., 2008; Zhang et al., 2016). An efficient alternative is to engineer SCN resistance genes into favorable cultivars.

In molecular genetics, the promoter is an important *cis*-regulatory elements to regulate transcription in plants. The promoter also determines the location and duration of transcript abundances. Thus far, a wide range of different promoters have been used in plant genetic engineering. Constitutive promoters are commonly used to drive transgene expression in transgenic engineering. Several constitutive promoters such as the *Cauliflower mosaic virus* 35S (CaMV 35S), maize ubiquitin-1, and rice actin-1 were used routinely to drive transgene expression in plants (De Block et al., 1987; Cao et al., 1992; Stewart et al., 1996; Nayak et al., 1997). Although constitutive promoters can be useful for increasing the expression of target genes, the strong expression of the target gene in all tissue types frequently leads to altered plant phenotype (Potenza et al., 2004; Gurr and Rushton, 2005; Pino et al., 2007). The high levels of transgene expression at all times cause an unnecessary loss of plant energy and increase the possibility of target gene silencing (Elmayan and Vaucheret, 1996; Breitler et al., 2004; Xia et al., 2010; Taha et al., 2012). In addition, strong constitutive promoters have limited value for developing multi-trait transgenic plants (Meyer and Saedler, 1996; Venter, 2007; Liu et al., 2014). These obstacles can be overcome by the use of tissue-or developmental stage-specific or inducible promoters to drive gene expression conditionally.

Many promoters have been shown to direct gene expression in selected tissues in response to specific stimuli from biotic and abiotic stresses (Bratić et al., 2009; Freeman et al., 2011; Ben Saad et al., 2020; Misra and Ganesan, 2021). In inducible defense mechanisms, plants recognize the presence of biotic or abiotic stresses *via cis*-acting elements within the promoter leading to the gene expression (Breitler et al., 2004). A variety of inducible promoters have been studied direct inducible gene expression in response to wounding, pathogen infection, and drought stress, which include the promoters of tomato lipoxygenase D (*TomLoxD*), potato wound-inducible 1 (*wun1*), potato proteinase inhibitor 2 (*pin2*), rice probenazole 1 (*pbz1*), and soybean responsive to dehydration 26 (*rd26*; Logemann et al., 1989; Xu et al., 1993; Mei et al., 2006; Yan et al., 2013; Freitas et al., 2019). These inducible promoters can be used to direct gene expression solely to the targeted tissues where expression of a specific gene is necessary.

Engineering genes for SCN resistance whose expression is directed by inducible promoters may be an attractive strategy. Target gene silencing in nematode feeding sites using SCN-inducible promoters are another tool to control SCN (Kandoth et al., 2011; Liu et al., 2012; Guo et al., 2015; Hewezi and Baum, 2015). To date, several nematode inducible promoters have been identified and functionally characterized in *Arabidopsis thaliana* plants and showed strong and controlled gene expression in targeted root tissues (Karczmarek et al., 2004; Hewezi et al., 2010, 2014, 2015; Siddique et al., 2011). Although several SCN-inducible promoters have been identified in soybean (Kandoth et al., 2011; Liu et al., 2012, 2014), the detailed functional characterization of SCN-inducible promoters in soybean plants is still limited. Therefore, considerable attention toward functional analysis of SCN-inducible promoters for specific expression of gene-of-interest in the targeted soybean cell types is needed. The construction of synthetic promoters using *cis*-regulatory motifs can potentially provide better control of gene expression (Liu and Stewart, 2016). Several studies have shown that synthetic promoters are able to enhance gene expression in a precise and predictable manner (Rushton et al., 2002; Bhullar et al., 2003; Venter, 2007; Liu et al., 2011; Zhang et al., 2019). The components for engineering synthetic promoters are products of *cis*-regulatory motifs in specific ways (Inaba et al., 2007; Liu et al., 2014; Zhang et al., 2019). These core regulatory elements can be utilized to design synthetic promoters with improved sensitivity and specificity (Liu and Stewart, 2016). The use of synthetic promoters with the combination of multiple *cis*-regulatory elements allows for the controlling multi-transgene expression (Liu and Stewart, 2016). Misra and Ganesan (2021) suggested that synthetic promoters are more effective in targeted gene expression compared to their native form. To date, many abiotic and biotic stress-inducible synthetic promoters have been developed and characterized in planta (Dey et al., 2015). However, engineering SCN-inducible synthetic promoters using *cis*-regulatory elements is currently limited.

Using bioinformatic tools, we previously identified cis-regulatory motifs in the soybean genome during soybean-SCN interaction (Liu et al., 2014). There, we discovered 116 overlapping SCN-inducible motifs among promoters of 18 co-expressed soybean genes during compatible interaction between soybean and SCN. Among them, a total of 11 motifs were shown to be SCN-inducible and identified 23 core motifs using the three best bioinformatic tools (SCOPE, W-AlignACE, and Weeder). The inducibility of 23 core motifs was evaluated in the transgenic hairy roots in the presence of SCN. Liu et al. (2014) selected two strong inducible motifs (M1.1 and M2.3) for further evaluation. The inducible motifs were used to develop synthetic promoters (Liu et al., 2014).

In the present study, we aimed to explore the functionality of the SCN-inducible synthetic promoters in whole plants by developing stable transgenic soybean plants. As an additional measure, we examined the specificity of the SCN-inducible synthetic promoters in response to various biotic and abiotic stresses. The present study provides insights into the potential applications of synthetic promoters for SCN resistance improvement in this economically important crop.

## Materials and methods

### Vector construction

Two SCN-inducible motifs (M1.1 and M2.3) were used from our previous study (Liu et al., 2014). The core motifs within these two synthetic promoters are listed in Supplementary Table S1. The 4× repeat of the M1.1 (TAAAATAAAGTTCTTTAATT) or M2.3 (ATATAATTAAGT) sequence were upstream of the minimal CaMV 35S promoter (−46 35S) and *GUS* reporter gene were used to develop SCN-inducible synthetic promoter constructs. Vector construction was carried out using binary vector pTF101.1 as the backbone. The pTF101.1 binary vector consisted of 4 × M1.1 or 4 × M2.3 promoter::*GUS*::Nos terminator cassette for reporter gene expression and 2 × 35S promoter::*Bar*::Nos terminator cassette for plant selection. These vector constructs were named as pTF101.1 (4 × M1.1) and pTF101.1 (4 × M2.3), respectively (Supplementary Figure S1). The positive control vector consisted of the CaMV 35S promoter instead of the synthetic promoter for constitutive reporter gene expression. The negative control vector was constructed by replacing the CaMV 35S promoter with the minimal −46 35S promoter (Supplementary Figure S1).

### Generation of transgenic plants

The binary vector constructs were transferred into *Agrobacterium tumefaciens* strain EHA101 by the heat-shock method. The binary constructs were introduced into soybean cv. “Williams 82” cotyledons by *Agrobacterium-*mediated transformation (Li et al., 2017). All plant material was cultured in a growth chamber (Percival Scientific Inc. Perry, IA, United States) at 24°C under a photoperiod of 16/8 h (light/dark) with 140 μmol/m^2^ s light intensity. Shoots were generated on a selective medium containing 6 mg/L glufosinate-ammonium. After rooting, the putative transgenic plantlets were transferred to Fafard 3B professional potting mix (Sun Gro Horticulture, Agawam, MA, United States). Transgenic T_0_ soybean plants were confirmed for the presence of transgene by painting Finale^®^ herbicide with glufosinate-ammonium as an active ingredient on the leaf surface. T_1_ progeny of the T_0_ individual lines was also confirmed using Finale^®^ herbicide with a segregation ratio of 3:1 (resistant: susceptible). T_2_ seeds were harvested from self-pollinated T_1_ progeny. T_2_ progeny were screened for herbicide selection and those that showed 100% resistance to Finale^®^ herbicide were selected. Independent homozygous T_3_ lines for each promoter construct were selected for further analysis. A chi-squared test was conducted to determine whether observed segregation ratios were significantly different from expected ratios.

### Plant growth conditions

Transgenic soybean plants (T_1_, T_2_, and T_3_ generations) were grown in 10 L pots containing potting mix and supplemented with Peter’s^®^ professional 20-20-20 general purpose fertilizer (An ICL Fertilizers Company, Dublin, OH, United States) in the greenhouse. The environmental conditions of the greenhouse were 16 h-light/8 h-dark photoperiod and 25°C temperature with fluctuations from a minimum of 22°C to a maximum of 28°C.

### Analysis of transgenic plants

Total genomic DNA was isolated from 1 g of fresh leaves of young 3-week-old plants using the CTAB extraction method (Stewart and Via, 1993). The insertion of the transgene (SCN-inducible synthetic promoter driving *GUS*) and (2 × 35S promoter driving *bar* gene) was confirmed by PCR using T_3_ transgenic soybean genomic DNA as a template (Supplementary Figures S2A,B). Genomic DNA was diluted to 100 ng/μl for PCR. PCR conditions for the *GUS* gene were as follows: 98°C for 2 min followed by 30 cycles at 98°C for 30 s, 60°C for 30 s, and 72°C for 30 s, and a final extension at 72°C for 5 min. PCR conditions for the *Bar* gene were as follows: 98°C for 2 min followed by 30 cycles at 98°C for 30 s, 64°C for 30 s, and 72°C for 30 s, and a final extension at 72°C for 5 min. The *Agrobacterium* contamination in transgenic lines was tested by confirming PCR using the primer set from the *Agrobacterium* backbone (Supplementary Figure S2C). To amplify the *chvA* (chromosomal virulence gene A) gene, as a control for the *Agrobacterium* contamination, the PCR conditions were as follows: 98°C for 2 min followed by 30 cycles at 98°C for 30 s, 65°C for 30 s, and 72°C for 30 s, and a final extension at 72°C for 5 min. PCR products were visualized on 0.8% agarose gels containing ethidium bromide. Primers used for the genotypic analysis of transgenic lines are provided in Supplementary Table S2.

### Gus expression analysis

Three independent T_3_ homozygous transgenic lines for each SCN-inducible promoter construct were used for GUS expression analysis. GUS histochemical assays were performed with the substrate 5-bromo-4-chloro-3-indoxyl-beta-D-glucuronide cyclohexylammonium salt (X-Gluc; Gold Biotechnology, St Louis, MO, United States). Plant tissue samples were harvested and soaked in GUS staining solution (2 mM X-Gluc, 50 mM potassium phosphate buffer, 5 mM potassium ferricyanide, 5 mM potassium ferrocyanide, and 0.2% Triton X-100; Jefferson, 1989). GUS staining was carried out overnight at 37°C. After GUS staining, tissues were cleared by replacing the GUS solution with 75% ethanol. GUS activity was quantified by fluorometric GUS analysis using 4-methylumbelliferyl-β-D-glucuronide hydrate (MUG; Sigma-Aldrich, St. Louis, MO, United States) as the substrate. The MUG assay (Jefferson, 1989) was conducted using 200 mg of ground tissue powder in 400 μl of ice-cold extraction buffer (50 mM of sodium phosphate buffer, NaHPO_4_ (pH 7.0), 1 mM of Na_2_EDTA, 10 mM DTT, 0.1% of Sodium Lauryl Sarcosine, 0.1% of Triton X-100). After adding the extraction buffer, the content was mixed with vortex and pipetting. Then the samples were centrifuged at 15,000 rpm for 10 min at 4°C. A 50 μl aliquot of tissue extracts was mixed with 500 μl of the prewarmed (37°C for 30 min) assay buffer (1 mM MUG in extraction buffer). The mixture was incubated at 37°C for 20 min and transferred 100 μl aliquot to the 900 μl stop buffer (0.2 M Na_2_CO_3_). After 20 min, the GUS activity was determined by measuring the fluorescent at 360 nm excitation and 444 nm emission using a Synergy H1 multi-detection microplate reader (Bio-Tek Instruments Inc., Santa Clara, CA, United States). Stop buffer and 0 to 80 nM 4-methylumbelliferone (4-MU; Sigma-Aldrich) were used for the standard curve. GUS activity was expressed as nM of 4-MU per minute per mg protein of total soluble protein. The protein concentration of each extract was determined using Qubit Protein Assay Kit (Thermo Fisher Scientific, Pittsburgh, PA, United States).

### Nematode source and inoculation

Root-knot nematode (RKN; *Meloidogyne incognita*) and SCN HG type 0 (race 3) were used for the nematode infection assays as previously described (Rambani et al., 2015). The second-stage juveniles (J2s) of the RKN and SCN were used as a source of inoculum. Transgenic T_3_ soybean seeds were surface sterilized with 10% sodium hypochlorite for 10 min followed by rinsing with deionized water for 30 min. The sterilized seeds were germinated on germination paper (Anchor Paper Co. Saint Paul, MN, United States) in the growth chamber at 27°C in the dark for 5 days. The germinated seedlings were placed on 150 mm blue blotter paper (Anchor Paper Co.) on a large petri plate (150 mm diameter). The blue blotter paper was dampened with 10 mM MES water (pH 6.5) before placing the seedling onto it. Each radicle of seedlings was inoculated with 500 J2s of the corresponding nematode. Inoculated radicles were covered with smaller blue blotter paper (70 mm diameter) and shoots of seedlings were exposed to light to ensure the growth of plants. Inoculated seedlings were grown in the growth chambers at 27°C (16 h-light/8 h-dark) for 12 days. The GUS staining was conducted when the gall started to appear in the RKN-inoculated roots, which was 8 days after inoculation (8 DAI). Then, the GUS staining was also performed after 12 DAI. Similar time points were also followed for SCN inoculation.

### Drought treatment

For drought stress, 3-week-old T_3_ homozygous transgenic soybean plants were subjected to water deprivation withholding water for 7 days. Transgenic plants without water deprivation were used as control.

### Salt treatment

For salt stress, 3-week-old T_3_ homozygous transgenic plants were irrigated with 250 mM NaCl solution. Transgenic plants were salt treated for 7 days. The transgenic plants without salt stress were used as control.

### Cold treatment

For cold stress, 3-week-old T_3_ homozygous transgenic plants were subjected to 4°C. Cold-treated samples were harvested at two different time points (6 and 24 h). The transgenic plants without cold stress were used as control.

### Wounding

Wounding was performed by cutting from the edges of 3-week-old T_3_ homozygous transgenic soybean leaves with scissors. The wounded tissues were harvested after 6 and 24 h. The transgenic plants without wounding were used as control.

### Phytohormone treatment

Three-week-old T_3_ homozygous transgenic soybean plants were treated with salicylic acid (SA), methyl jasmonate (MeJA), and abscisic acid (ABA). Plant roots were washed gently with water to remove potting mix and then were soaked into 200 ml solutions with 100 μM SA, MeJA, or ABA. The transgenic plants were soaked in water as a control. Control and phytohormone-treated samples were collected at 6 and 24 h post treatments.

### Insect herbivore treatment

The inducibility of synthetic promoters was tested in insect-treated plants. First-instar larvae of fall armyworm (*Spodoptera frugiperda*) were introduced on the surface of leaves of 3-week-old T_3_ homozygous transgenic plants. Each transgenic plant was placed on a plastic tray covered with a dome-shaped plastic lid and infested with five larvae on each plant. The insect-treated plants were kept in the growth chamber under 16/8 h (light/dark) at 25°C. Transgenic plants without insect treatments were used as control. Three independent transgenic lines from each promoter construct and six plants from each transgenic line were used as a biological control for the experiment. The whole plant assay was performed at three time points, 24, 48, and 72 h.

### Bacterial pathogen treatment

Three-week-old T_3_ homozygous transgenic soybean plants were tested with bacterial pathogens (*Pseudomonas syringe* pv. *glycinea*, *P. syringe* pv. *tomato*, and *P. marginalis*) provided by Dr. Bonnie Ownley (University of Tennessee, Knoxville, TN, United States). For bacterial inoculation, the strains were grown in tryptic soy broth (pancreatic digest of casein 17.0 g/L, papaic digest of soybean 3.0 g/L, dextrose 2.5 g/L, sodium chloride 5.0 g/L, and dipotassium phosphate 2.5 g/L) at 28°C with shaking at 225 rpm. The liquid cultures were spun at 3000 rpm for 10 min for collecting pelleted cells. The cells were dissolved in 10 mM MgCl_2_ to an optical density OD_600_ of 0.3. The plants were immersed into bacterial solution and placed into a 20 L vacuum chamber (Best Value Vacs, Naperville, IL, United States). A vacuum pressure of ~−90 kPa was applied for 30 min three times, with regular agitation of bacterial solution while plants were submerged. For mock control treatments, a 10 mM MgCl_2_ solution was used for the vacuum infiltration of plants. After infiltration, the plants were kept in a closed container with high humidity. Three independent transgenic lines from each promoter and six plants from each transgenic line were used for the experiment.

### RNA extraction and qRT-PCR

TRIzol™ reagent (Thermo Fisher Scientific) was used to extract the total RNA from treated and untreated leaves and root samples according to the manufacturer’s protocol. The total RNA was quantified using a NanoDrop™ spectrophotometer ND-1000 (Thermo Fisher Scientific) and the RNA integrity was checked by agarose gel electrophoresis. The total RNA was treated with DNaseI, and column purified with the RNA Clean & Concentrator™ kit (Zymo Research, Foster City, CA, United States) to remove genomic DNA contamination. Total RNA (1 μg) was used to synthesize first-strand cDNA in a 20 μl reaction volume containing 1 μl of 50 μM oligo dT primer and 1 μl of 10 mM dNTP mix, 2 μl of 10 × RT buffer, 4 μl of 25 mM MgCl2, 2 μl of 0.1 M DTT, 1 μl of RNaseOUT™ (40 U/μl), and 1 μl of SuperScript^®^ III RT (200 U/μl). The GUS gene-specific primers (Supplementary Table S2) were designed for qRT-PCR using Primer3 (v 0.4.0). Real-time PCR was conducted in a 15 μl reaction volume containing, 7.5 μl of Power SYBR Green 2X Master Mix (Applied Biosystems, Foster City, CA, United States), 1 μl of cDNA (12.5 ng), 0.375 μl of each primer (10 μM), and 5.75 μl of H_2_O. The real-time PCR was carried out on a QuantStudio™ 6 Flex Real-Time PCR System (Applied Biosystems). The results were analyzed using a standard curve method for relative expression normalized to the soybean ubiquitin gene (*GmUBI3*).

## Results

### Analysis of the synthetic promoters in transgenic soybean plants

Six independent transgenic T_1_ soybean lines were generated for each synthetic promoter construct 4 × M1.1 and 4 × M2.3 and four lines for each construct were confirmed to contain a single T-DNA insertion using herbicide-resistance segregation analysis. Subsequently, homozygous T_3_ transgenic soybean lines were also screened by PCR for the presence of transgenes *GUS* and *Bar* (Supplementary Figures S2A,B). Three independent stable transgenic soybean (T_3_ homozygous) lines were selected for each promoter construct to conduct further analysis.

### Unstressed conditions

**Figure 1:**
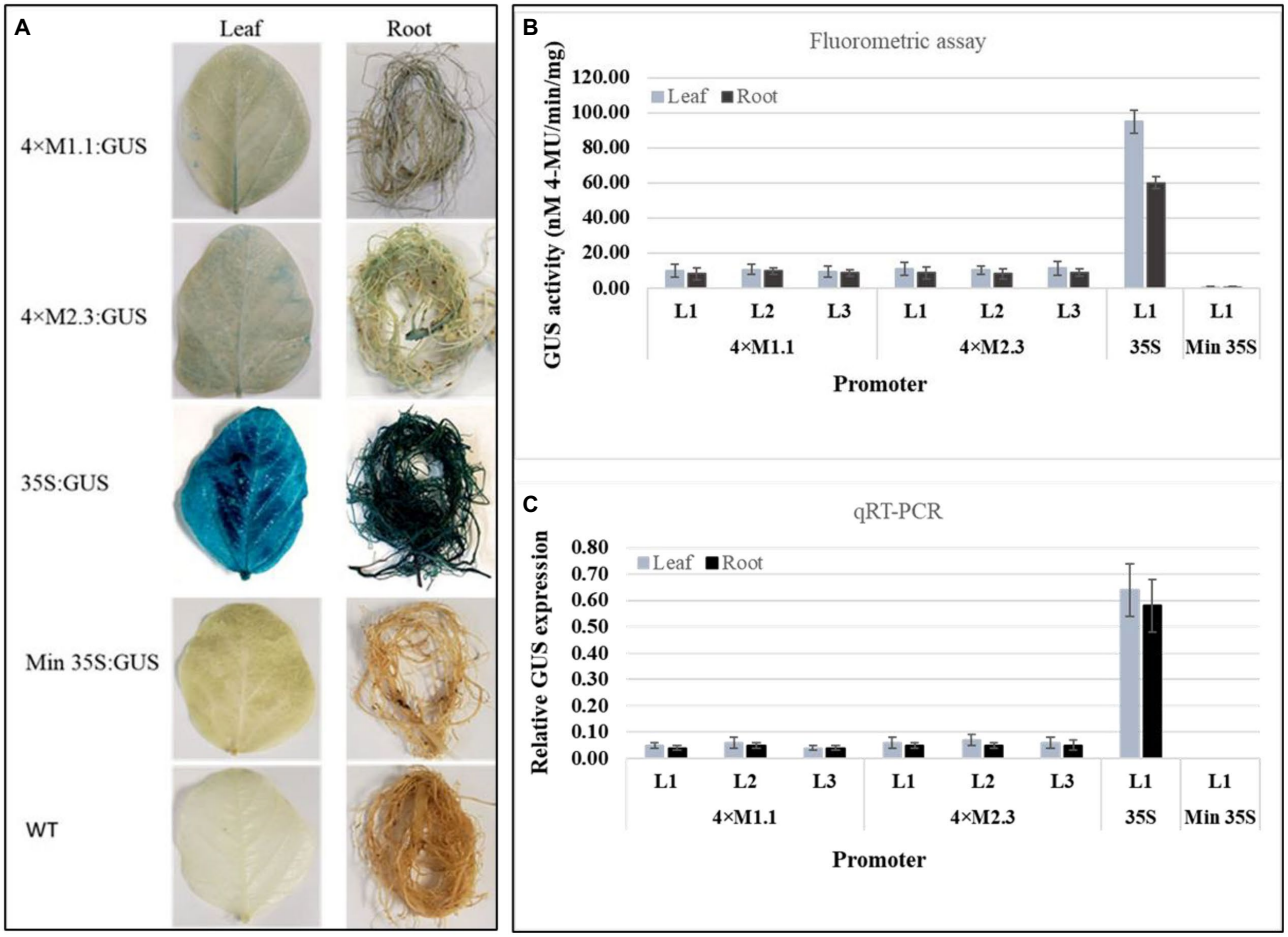
Basal GUS activity in leaves and roots of 3-week-old T_3_ transgenic soybean containing the individual promoter-GUS construct under unstressed condition. **(A)** Histochemical staining for GUS activity in transgenic soybean and wild-type (WT) plants. **(B)** Fluorometric assay for GUS activity in transgenic soybean plants. **(C)** Quantitative real-time RT-PCR (qRT-PCR) analysis for GUS expression in transgenic soybean plants. The relative levels of transcripts were normalized to soybean ubiquitin gene (*GmUBI*3). Three independent transgenic lines (L1, L2, and L3) were used for 4 × M1.1 and 4 × M2.3 promoter-GUS constructs. One transgenic line (L1) was used for 35S and minimal (Min) 35S promoter-GUS constructs. Bars represent mean values of six biological replicates ± standard error.

The basal level of GUS activity in the transgenic soybean plants containing the individual synthetic promoters (4 × M1.1 and 4 × M2.3) was assessed to determine the functionality of the synthetic promoters in whole plants. Histochemical GUS staining showed very weak GUS expression in leaves and roots of the transgenic plants containing either 4 × M1.1::GUS or 4 × M2.3::GUS construct, similarly no GUS staining was observed in transgenic plants containing the minimal 35S promoter and in the wild-type plants (Figure 1A). Very strong GUS staining was observed in leaves and roots of the transgenic plants containing 35S promoter (Figure 1A). For the quantitative GUS expression analysis, fluorometric GUS assay and qRT-PCR were conducted using three independent transgenic soybean lines (L1, L2, and L3) for each synthetic promoter (Figures 1B,C). Fluorometric GUS assays showed very weak GUS activity in the leaves and roots (Figure 1B). Similarly, the qRT-PCR analyses indicated very low level of GUS transcript abundance in the leaves and roots (Figure 1C). There were no significant differences in GUS activity among transgenic lines of each synthetic promoter (Figures 1B,C). No GUS activity was detected in transgenic plants containing the minimal 35S promoter, whereas strong GUS activity was detected in the transgenic plants containing 35S promoter (Figures 1B,C). These results indicated that both qualitative histochemical GUS staining and quantitative GUS analyses were consistent for each synthetic promoter with a low background level of GUS activity in the leaves and roots of transgenic soybean plants.

### Nematode-infected plants

#### Soybean cyst nematode

**Figure 2:**
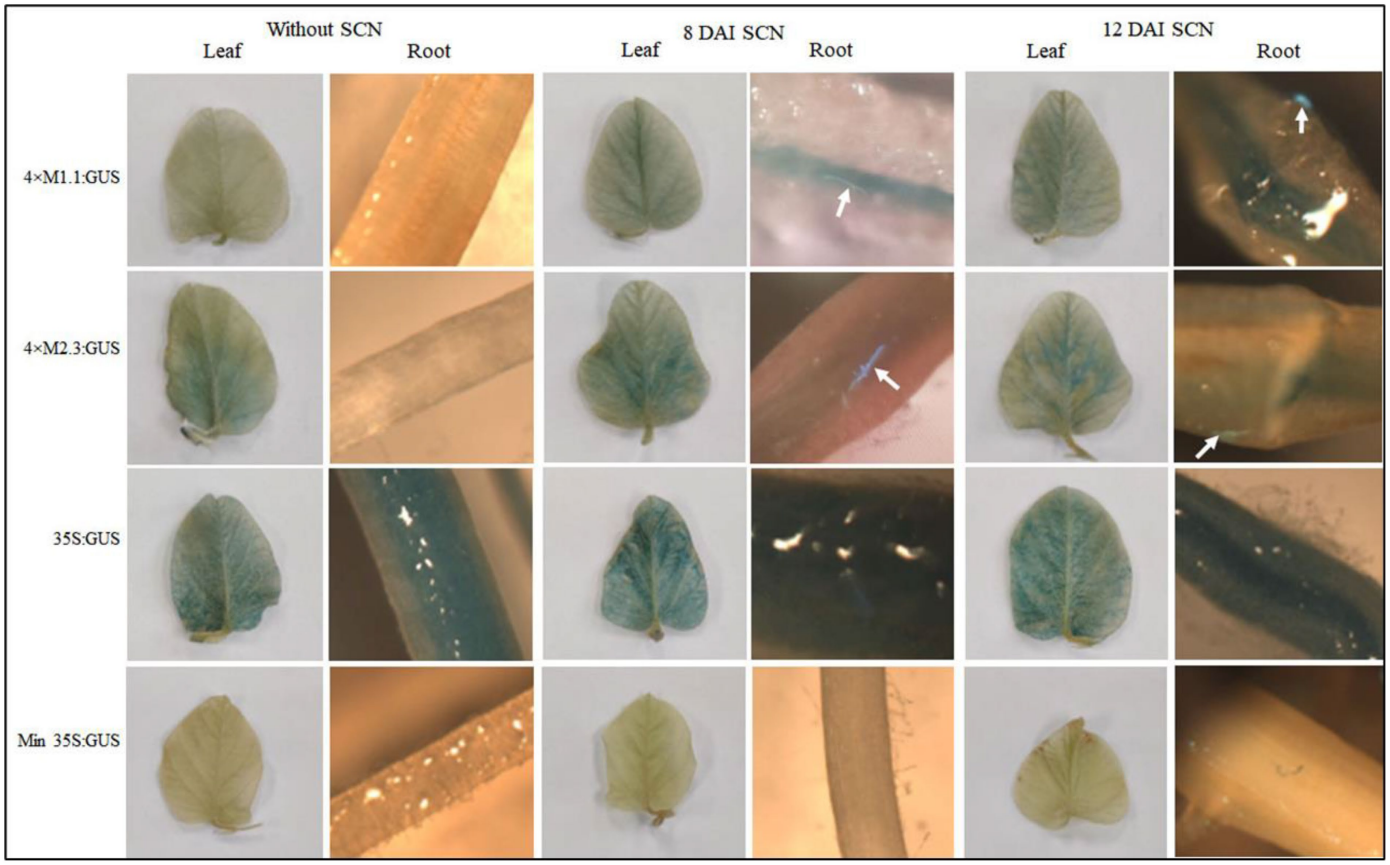
Histochemical GUS activity in leaves and roots of transgenic soybean containing the individual promoter-GUS construct uninfected or infected with soybean cyst nematode (SCN, *Heterodera glycines*) at 8 and 12 days after inoculation (DAI). Arrows indicate localized GUS expression within the nematode-induced feeding sites. Three independent transgenic lines and six plants from each line were used for 4 × M1.1 and 4 × M2.3 promoter-GUS constructs. One transgenic line and six plants from the transgenic line were used for 35S and minimal (Min) 35S promoter-GUS constructs. A similar GUS expression was observed from each line.

The activity of each synthetic promoters in transgenic soybean plants was analyzed at 8 and 12 DAI with SCN (*H. glycines*). The transgenic soybean plants containing 4 × M1.1 promoter had increased GUS activity in the nematode-infected roots at 8 DAI. The GUS activity was strongly induced at 12 DAI compared to the non-infected roots (Figure 2). The transgenic soybean plants containing 4 × M2.3 promoter also showed increased GUS activity in the nematode-infected roots at both 8 and 12 DAI, with more intense GUS activity at 12 DAI (Figure 2). No induced GUS activity was observed in non-infected roots. Furthermore, there was no detectable GUS activity in the leaves of the transgenic plants infected with nematode for both synthetic promoters (Figure 2). The 35S promoter endowed strong and consistent GUS activity in both roots and leaves of infected and non-infected transgenic plants. No detectable GUS expression was found in the leaves and roots of infected and non-infected transgenic plants containing the minimal 35S promoter (Figure 2). These results suggest that the synthetic promoters were activated after SCN infection specifically at the nematode feeding sites.

#### Root-knot nematode

**Figure 3:**
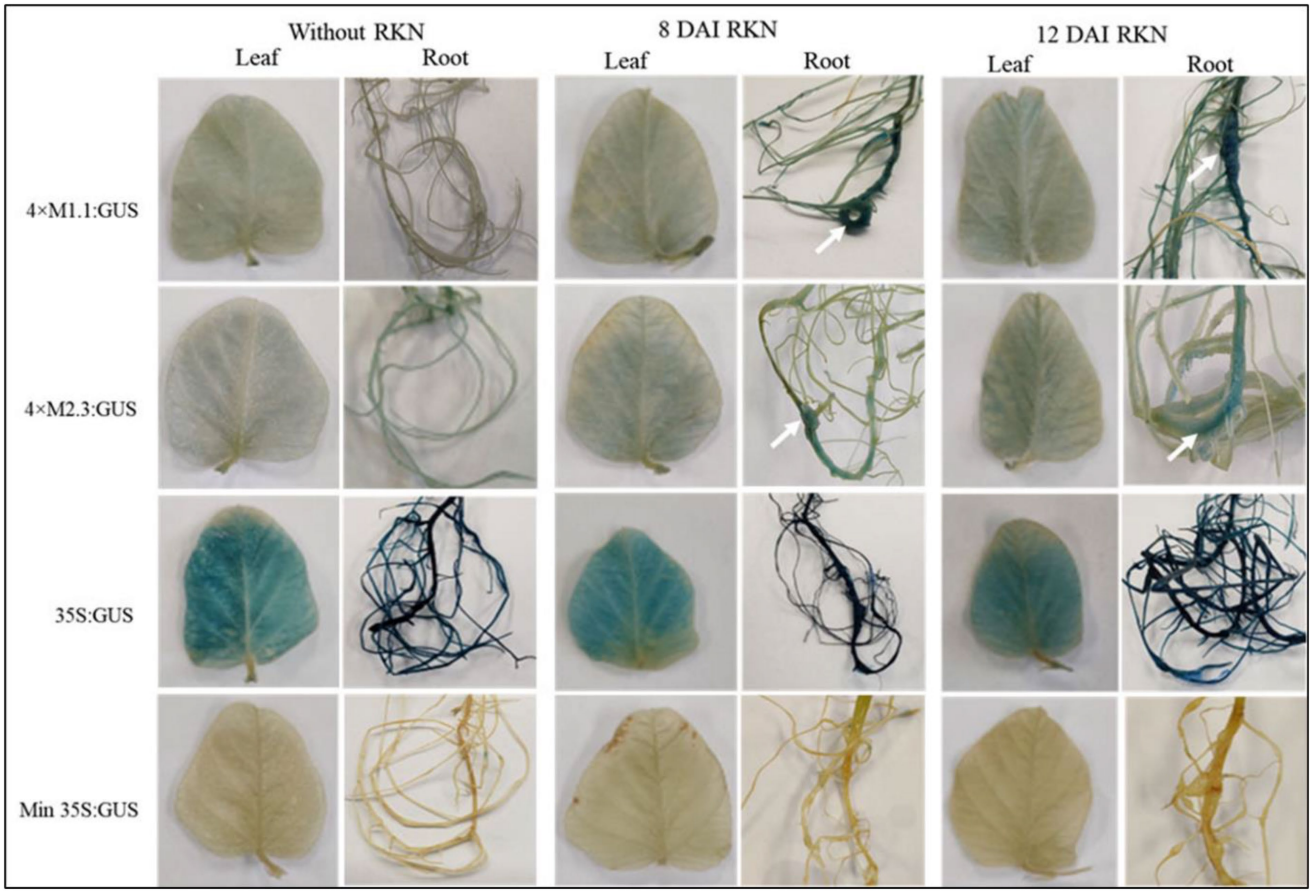
Histochemical GUS activity in leaves and roots of transgenic soybean containing the individual promoter-GUS construct uninfected or infected with root-knot nematode (RKN, *Meloidogyne incognita*) at 8 and 12 days after inoculation (DAI). Arrows indicate localized GUS expression within the nematode-induced galls. Three independent transgenic lines and six plants from each line were used for 4 × M1.1 and 4 × M2.3 promoter-GUS constructs. One transgenic line and six plants from the transgenic line were used for 35S and minimal (Min) 35S promoter-GUS constructs. A similar GUS expression was observed from each line.

Transgenic soybean plants containing individual synthetic promoters were analyzed for GUS activity at 8 and 12 DAI with RKN (*M. incognita*). After nematode infection, both synthetic promoters (4 × M1.1 and 4 × M2.3) were strongly induced in the nematode-induced galls at 8 and 12 DAI compared to non-infected roots (Figure 3). The 4 × M1.1 promoter showed stronger GUS activity at both time points compared to the 4 × M2.3 promoter (Figure 3). There was no GUS activity in the leaves of the transgenic plants infected with *M. incognita* for both the synthetic promoters (Figure 3). The patterns of GUS staining under the control of 35S promoter were comparable between nematode-infected and non-infected roots. No GUS activity was observed in the roots and leaves under the control of the minimal 35S promoter (Figure 3). These results suggest that the synthetic promoters were activated after RKN infection specifically in the nematode-induced galls.

### Abiotic stressed condition

#### Drought

**Figure 4:**
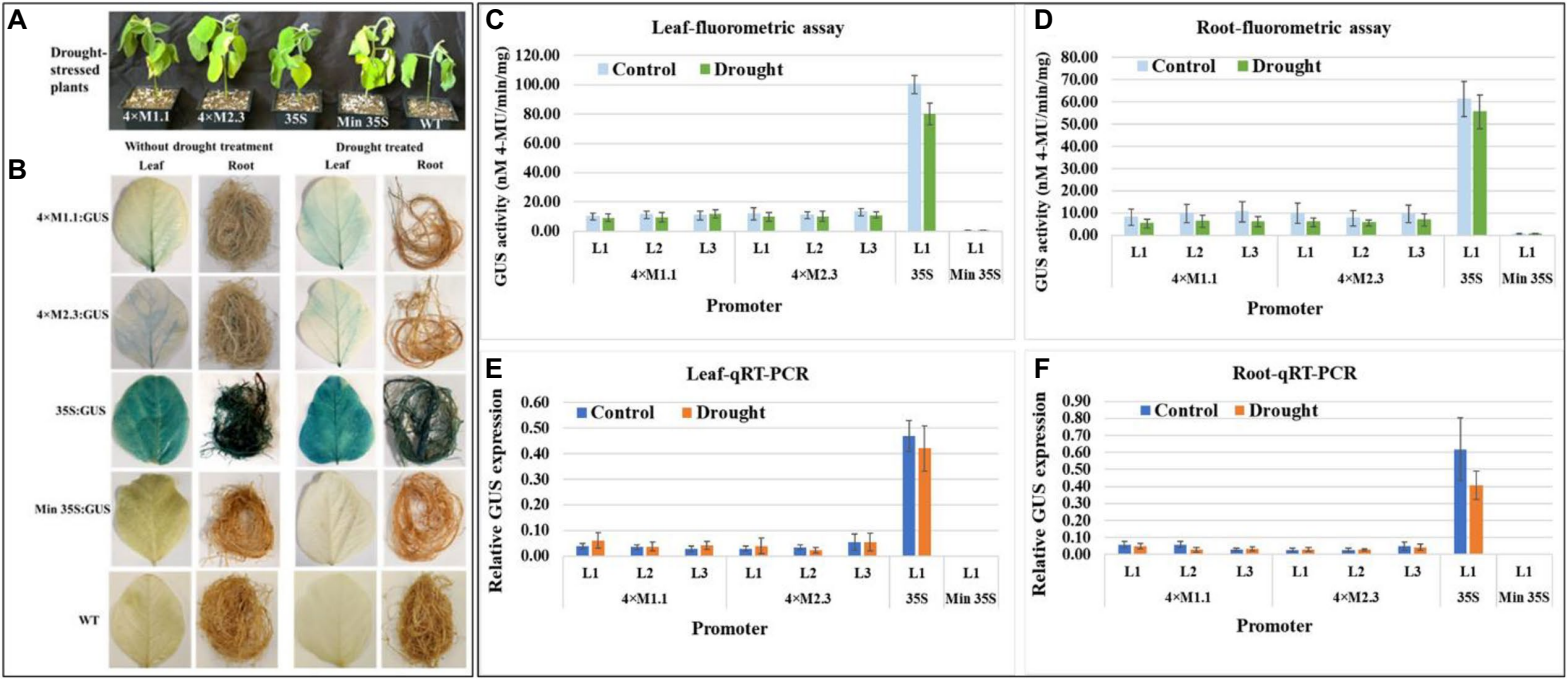
GUS activity in leaves and roots of 3-week-old T_3_ transgenic soybean containing the individual promoter-GUS construct subjected to the absence (untreated control) and presence (treated) of drought. **(A)** Representative plants were subjected to dehydration condition *via* water deprivation for 7 days. **(B)** Histochemical staining for GUS activity in transgenic soybean and wild-type (WT) plants. **(C,D)** Fluorometric assay for GUS activity in leaf **(C)** and root **(D)**. **(E,F)** Quantitative real-time RT-PCR (qRT-PCR) analysis for GUS expression in leaf **(E)** and root **(F)**. The relative levels of transcripts in qRT-PCR were normalized to soybean ubiquitin gene (*GmUBI*3). Three independent transgenic lines (L1, L2, and L3) were used for 4 × M1.1 and 4 × M2.3 promoter-GUS constructs. One transgenic line (L1) was used for 35S and minimal (Min) 35S promoter-GUS constructs. Bars represent mean values of six biological replicates ± standard error. Statistical analysis by a two-sample paired *t*-test (*p* < 0.05) indicated no significant differences between treated and untreated plants.

The effect of drought stress on GUS expression driven by the synthetic promoters in the transgenic plants was examined. The plants were subjected to dehydration *via* water deprivation for 7 days (Figure 4A) before assaying GUS activity. Histochemical GUS staining of the leaves and roots of the transgenic plants showed no detectable GUS induction compared to non-treated tissues for both synthetic promoters (4 × M1.1 and 4 × M2.3; Figure 4B). The 35S promoter line showed intense GUS staining in both treated and untreated tissues, whereas no GUS staining was detected in the minimal 35S promoter line and wild-type soybean plants (Figure 4B). Fluorometric GUS assays indicated no significant changes in GUS expression between drought-treated and non-treated leaves and roots of transgenic plants for each synthetic promoter (Figures 4C,D). Similarly, qRT-PCR analyses showed that there were no significant changes in *GUS* expression between drought-treated and non-treated leaves and roots of transgenic plants for both synthetic promoters (Figures 4E,F). These results suggested that the synthetic promoters were not activated in response to drought stress.

#### Salt

**Figure 5:**
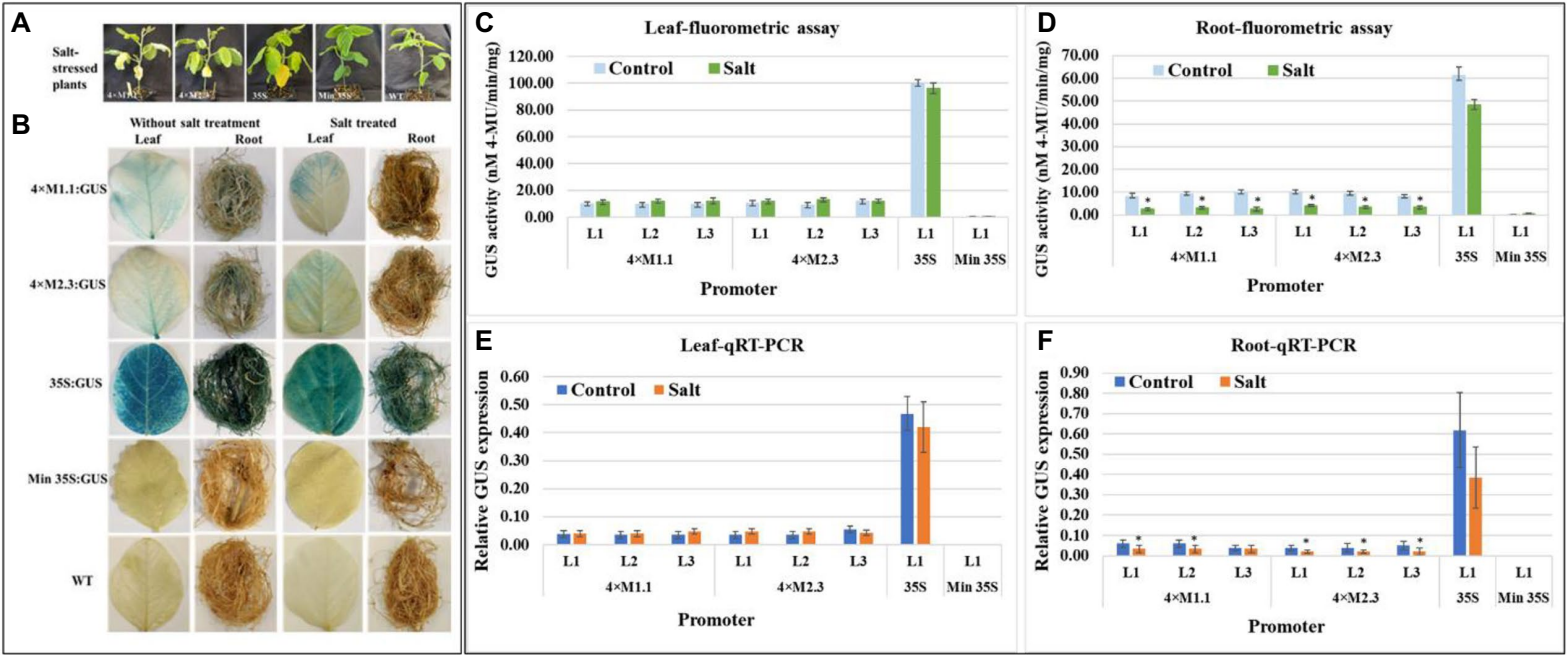
GUS activity in leaves and roots of 3-week-old T_3_ transgenic soybean containing the individual promoter-GUS construct subjected to mock (untreated control) and salt treatment. **(A)** Representative plants were irrigated with 250 mM NaCl solution for 7 days. **(B)** Histochemical staining for GUS activity in transgenic soybean and wild-type (WT) plants. **(C,D)** Fluorometric assay for GUS activity in leaf **(C)** and root **(D)**. **(E,F)** Quantitative real-time RT-PCR (qRT-PCR) analysis for GUS expression in leaf **(E)** and root **(F)**. The relative levels of transcripts in qRT-PCR were normalized to soybean ubiquitin gene (*GmUBI*3). Three independent transgenic lines (L1, L2, and L3) were used for 4 × M1.1 and 4 × M2.3 promoter-GUS constructs. One transgenic line (L1) was used for 35S and minimal (Min) 35S promoter-GUS constructs. Bars represent mean values of six biological replicates ± standard error. Statistical analysis by a two-sample paired *t*-test (*p* < 0.05) indicated no significant differences between treated and untreated plants.

For salt stress, plants were treated with 250 mM NaCl solution for 7 days, and then evaluated for GUS activity (Figure 5A). The histochemical staining showed weak GUS activity in leaves for each synthetic promoter after the salt treatment (Figure 5B). The quantitative fluorometric GUS activity also showed no statistically significant differences in leaves compared to non-treated tissues (Figures 5C,E). Yet, GUS activity was lower in roots after the salt treatment compared to the non-treated tissues (Figures 5D,F). On average the GUS activity was 3.3-fold lower in roots driven by the 4 × M1.1 promoter and 2.5-fold lower in roots driven by the 4 × M2.3 promoter compared to non-treated roots (Figure 5D). Also, in qRT-PCR, the relative *GUS* expression level was 1.5-fold lower driven by the 4 × M1.1 promoter and 2-fold lower driven by the 4 × M2.3 promoter compared to the non-treated root tissues (Figure 5F). These results demonstrated that the synthetic promoters were not induced by the salt-stress condition.

#### Cold

**Figure 6:**
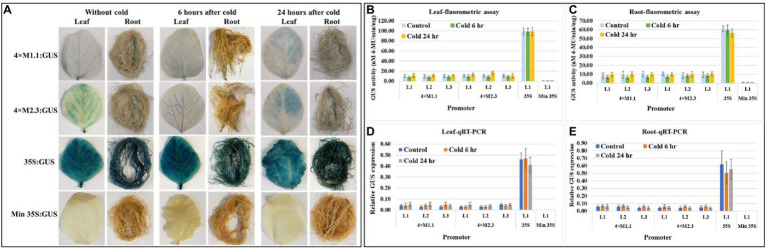
GUS activity in leaves and roots of 3-week-old T_3_ transgenic soybean containing the individual promoter-GUS construct subjected to the absence (untreated control) and presence (treated) of cold (4°C) at two time points of 6 and 24 h after treatment. **(A)** Histochemical staining for GUS activity in transgenic soybean plants. **(B,C)** Fluorometric assay for GUS activity in leaf **(B)** and root **(C)**. **(D,E)** Quantitative real-time RT-PCR (qRT-PCR) analysis for GUS expression in leaf **(D)** and root **(E)**. The relative levels of transcripts in qRT-PCR were normalized to soybean ubiquitin gene (*GmUBI*3). Three independent transgenic lines (L1, L2, and L3) were used for 4 × M1.1 and 4 × M2.3 promoter-GUS constructs. One transgenic line (L1) was used for 35S and minimal (Min) 35S promoter-GUS constructs. Bars represent mean values of six biological replicates ± standard error. Statistical significance (*p* < 0.05) was determined by two-sample paired *t*-test. Bars with asterisk (*) indicate significant difference compared to untreated control plants.

Histochemical GUS staining in cold stressed leaves showed no induced expression at 6 h after treatment but showed weak induction of GUS staining at 24 h after treatment compared to non-treated tissues (Figure 6A). No induced GUS staining was detected between cold-treated and non-treated root tissue at the two time points (Figure 6A). The quantitative fluorometric GUS activity showed no differences between treated and non-treated tissues for both synthetic promoters (4 × M1.1 and 4 × M2.3; Figures 6B,C). Similarly, the relative *GUS* transcript abundance in qRT-PCR reflected the fluorometric GUS activity (Figures 6D,E). The 35S promoter line showed strong and consistent GUS activity in leaves and roots of the cold and non-treated conditions (Figures 6A–E). No GUS activity was detected in transgenic plants containing minimal 35S promoter and in wild-type plants, regardless of cold-treated and non-treated tissues (Figures 6A–E).

#### Wounding

**Figure 7:**
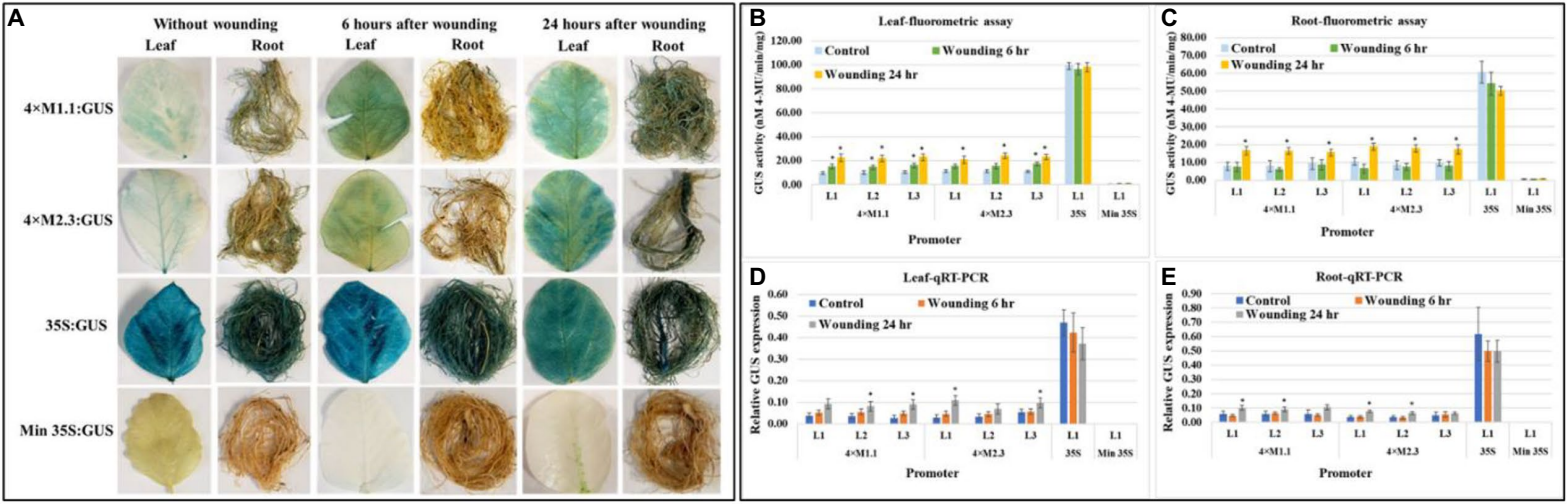
GUS activity in leaves and roots of 3-week-old T_3_ transgenic soybean containing the individual promoter-GUS construct subjected to the absence (untreated control) and presence (treated) of wounding at two time points of 6 and 24 h after treatment. **(A)** Histochemical staining for GUS activity in transgenic soybean plants. **(B,C)** Fluorometric assay for GUS activity in leaf **(B)** and root **(C)**. **(D,E)** Quantitative real-time RT-PCR (qRT-PCR) analysis for GUS expression in leaf **(D)** and root **(E)**. The relative levels of transcripts in qRT-PCR were normalized to soybean ubiquitin gene (*GmUBI*3). Three independent transgenic lines (L1, L2, and L3) were used for 4 × M1.1 and 4 × M2.3 promoter-GUS constructs. One transgenic line (L1) was used for 35S and minimal (Min) 35S promoter-GUS constructs. Bars represent mean values of six biological replicates ± standard error. Statistical significance (*p* < 0.05) was determined by two-sample paired *t*-test. Bars with asterisk (*) indicate significant difference compared to untreated control plants.

Histochemical GUS staining of wounded leaves showed a strong GUS induction for both the synthetic promoters (4 × M1.1 and 4 × M2.3) after 6 and 24 h treatment compared to unwounded leaves (Figure 7A). In roots, the GUS staining was not detected after 6 h, but induced GUS activity was observed after 24 h compared to untreated tissues (Figure 7A). Strong and consistent GUS staining was observed under the control of 35S promoter for both wounded and unwounded tissues. No GUS staining was seen under the control of the minimal 35S promoter in the wounded and unwounded tissues (Figure 7A). Fluorometric GUS assay of the leaf tissue showed stronger GUS activity at 6 and 24 h after wounding in all lines of both synthetic promoters except in lines (L1 and L2) of the 4 × M2.3 promoter (Figure 7B). Both the synthetic promoters showed an average of 1.5-fold increased GUS activity after 6 h of wounding and 2-fold increased after 24 h of wounding compared to the unwounded leaves (Figure 7B). In roots, increased GUS activity of about 2-fold was observed after 24 h of wounding in all lines of both synthetic promoters compared to unwounded tissues (Figure 7C). The qRT-PCR relative transcript abundance of GUS showed strong activity in leaves for 4 × M1.1 promoter lines L2 (2.3-fold increase), L3 (3.1-fold increase) and for 4 × M2.3 promoter lines L1 (3.7-fold increase), and L3 (1.7-fold increase) after 24 h of wounding (Figure 7D). In roots, the relative GUS expression level was induced only in the 4 × M2.3 promoter lines L1 (2-fold increase) and L2 (1.7-fold increase) compared to unwounded condition (Figure 7E).

### Phytohormones

#### Salicylic acid

There were no differences in the GUS histochemical staining driven by each synthetic promoter (4 × M1.1 and 4 × M2.3) between SA treated and non-treated leaf and root tissues in histological GUS staining (Supplementary Figure S3A). Consistently, fluorometric GUS assay and qRT-PCR analyses showed similar results for GUS expression in SA treated and non-treated leaves and root tissues for each synthetic promoter (Supplementary Figures S3B–E).

#### Methyl jasmonate

Both synthetic promoters (4 × M1.1 and 4 × M2.3) resulted in relatively strong GUS staining in leaves after 24 h of MeJA treatment compared to non-treated tissues in histochemical GUS staining (Supplementary Figure S4A). While no induced GUS staining was observed in the leaves after 6 h of treatment compared to non-treated tissues (Supplementary Figure S4A). In contrast to the leaf staining patterns, no induction of GUS was observed in the roots under the control of each synthetic promoter (Supplementary Figure S4A). Fluorometric GUS assays showed significant GUS expression in the leaves of lines L2 (1.6-fold increase) and L3 (1.8-fold increase) driven by the 4 × M1.1 promoter and an average 2-fold increase under the control of the 4 × M2.3 promoter after 24 h of treatment (Supplementary Figure S4B). Based on the fluorometric GUS assays and qRT-PCR analyses, there was no activation of the synthetic promoters in the roots compared to non-treated tissues (Supplementary Figures S4C,E).

#### Abscisic acid

Histochemical staining showed strong GUS expression in the leaves for both synthetic promoters (4 × M1.1 and 4 × M2.3) after 24 h of ABA treatment compared to the non-treated (Supplementary Figure S5A). However, the induction of promoter was not observed in the leaves after 6 h of treatment (Supplementary Figure S5A). In roots, very weak GUS staining was observed under each synthetic promoter after 24 h of ABA treatment (Supplementary Figure S5A). Fluorometric GUS assay showed stronger GUS activity in the leaves with an average of 1.8-fold higher under the control of the 4 × M1.1 promoter and an average of 2.7-fold higher under the control of the 4 × M2.3 promoter (Supplementary Figure S5B). The qRT-PCR GUS expression levels in the leaves were also consistent with the results of the fluorometric GUS assay (Supplementary Figure S5D). However, there were no differences in GUS activity in the roots (Supplementary Figure S5C). Gene expression quantification also showed no differences in GUS expression in the roots (Supplementary Figures S5D,E). These results suggest that the synthetic promoters were not activated in the roots, while were induced in the leaves in response to ABA treatment.

### Biotic stressed condition

#### Insect herbivore treatment

The activity of each synthetic promoters in transgenic soybean plants was analyzed at 24, 48, and 72 h after application of herbivory by fall armyworm (*S. frugiperda*) larvae. Histochemical GUS staining showed no induced GUS expression in the leaves and roots driven by each synthetic promoter (4 × M1.1 and 4 × M2.3) at the three time points after insect feeding (Supplementary Figure S6A). The further validation by fluorometric GUS assay and qRT-PCR analyses also showed no induced GUS expression compared to control tissues (Supplementary Figures S6B–E).

### Bacterial treatment

#### *Pseudomonas syringe* pv. *glycinea*

Histochemical GUS staining of the leaves infected by this soybean pathogenic bacterium species resulted in no induced GUS activity after 24 h of treatment for each synthetic promoter (4 × M1.1 and 4 × M2.3; Supplementary Figure S7A). However, the infected leaves had stronger GUS activity after 48 h of treatment for each synthetic promoter compared to non-infected tissues (Supplementary Figure S7A). Surprisingly, the increased GUS activity was abolished after 72 h of treatment for each synthetic promoter (Supplementary Figure S7A). No detectable GUS staining was observed in the roots under each synthetic promoter at any time points compared to non-infected root tissues (Supplementary Figure S7A). Similarly, fluorometric GUS assay indicated the induction of GUS activity in the leaves after 48 h of treatments for each synthetic promoter (Supplementary Figure S7B). The 4 × M1.1 promoter induced about 2-fold and the 4 × M2.3 promoter induced 1.5-fold higher GUS activity compared to the non-infected leaves (Supplementary Figure S7B). Also, no increased GUS activity was observed in the roots compared to the non-infected tissues (Supplementary Figure S7C). The relative qRT-PCR transcripts showed no significant GUS activity between infected and non-infected tissues (Supplementary Figures S7D,E). The patterns of GUS activity were consistent with the 35S promoter among infected and non-infected tissues. No significant GUS activity was detected in the leaves and roots under the control of the minimal 35S promoter construct (Supplementary Figures S7A–E).

#### *Pseudomonas syringe* pv. *tomato*

The intensity of GUS staining was relatively similar in the leaves and roots between infected and non-infected tissues corresponding to each synthetic promoter (4 × M1.1 and 4 × M2.3; Supplementary Figure S8A). The GUS expression driven by the 4 × M2.3 promoter showed the varied intensity of GUS in leaves only after 48 h of treatment (Supplementary Figure S8A). No induced GUS activity was detected in the leaves and roots in fluorometric GUS assay and qRT-PCR analyses (Supplementary Figures S8B–E). The GUS activity was consistent among infected and non-infected tissues containing 35S promoter. No significant GUS activity was detected in the leaves and roots under the control of the minimal 35S promoter construct (Supplementary Figures S8B–E).

#### Pseudomonas marginalis

Histochemical GUS staining showed no induced activity in the leaves and roots under the control of each synthetic promoter (4 × M1.1 and 4 × M2.3; Supplementary Figure S9A). Similar observations were made in fluorometric GUS assay and qRT-PCR analyses, where no induction of GUS activity was evident in leaves and roots compared with non-infected tissues (Supplementary Figures S9B–E). For 35S promoter, GUS expression was consistent between infected and non-infected tissues. No significant GUS activity was detected in the leaves and roots under the control of the minimal 35S promoter construct (Supplementary Figures S9B–E).

## Discussion

One of the challenges in engineering genes in plants is controlling the spatiotemporal transgene expression (Venter, 2007; Liu et al., 2013; Huang et al., 2021). The expression of transgene is largely dependent on the selection of precise promoter. Tissue-specific promoters can activate gene expression in certain cell types and improve gene expression spatially and temporally. *Via* bioinformatic and experimental approaches, we previously identified two regulatory motifs (M1.1 and M2.3) in the soybean genome during soybean-SCN interaction and developed synthetic promoters for inducibility in response to SCN infection using transgenic soybean hairy root system (Liu et al., 2014). The present study describes the functionality of these SCN-inducible synthetic promoters in whole transgenic soybean plants.

In transgenic soybean, both SCN-inducible synthetic promoters 4 × M1.1 and 4 × M2.3 were strongly activated within the nematode feeding structures induced by either SCN or RKN (Figures 2, 3). While the synthetic promoters directed GUS expression specifically to roots in the nematode-infected plants, no GUS activity was detected in leaves and roots of non-infected plants (Figures 2, 3). These results indicate that the synthetic promoters are nematode-responsive, particularly in the nematode-induced feeding structures, which makes them valuable as tools for developing efficient SCN or RKN resistance. Nematode-inducible promoters have potential to improve the control management through plant genetic engineering. For example, host-induced gene silencing by targeting conserved nematode parasitism gene led to effective control against plant-parasitic nematodes (Huang et al., 2006; Sindhu et al., 2009; Koch and Kogel, 2014; Hewezi and Baum, 2015). These types of studies can be conducted using our synthetic promoters, which will efficiently produce dsRNA in the feeding sites for SCN and RKN gene silencing.

Yet, the 4 × M1.1 promoter showed much higher GUS activity compared to 4 × M2.3 in the nematode-infected roots (Figures 2, 3). It may important to mention that this promoter contain the auxin-responsive cis-element TAAAGT (Liu et al., 2014). Auxin-responsive cis-elements play a fundamental role in the activation of promoters within SCN and RKN feeding sites (Wang et al., 2007; Hewezi et al., 2014). These findings might be associated with the observed higher induction of 4 × M1.1 promoter in the nematode-induced feeding structures.

Although our SCN-inducible synthetic promoters were originally developed from soybean-SCN interaction (Liu et al., 2014), the current study showed that these promoters were also inducible in response to RKN infection. These results may suggest that both the synthetic promoters contain conserved cis-acting elements responsive to SCN and RKN infection, taking into consideration the striking similarity between these two nematode species in infection processes.

Specificity of these synthetic promoters to nematode infection is considered as additional measures for their potential application in plant genetic engineering. Neither 4 × M1.1 promoter nor 4 × M2.3 promoter resulted in significant induction of GUS activity compared to the control under drought, salt, and cold conditions (Figures 4–6). On contrary, a reduced GUS activity in salt-stressed roots was observed (Figure 5). Salt stress severely compromises growth and development affecting the plant survival. Gene expression is largely impacted in roots under salt stress condition (Kawasaki et al., 2001; Nefissi Ouertani et al., 2021). Nevertheless, our results showed that these synthetic promoters were not inducible in response to drought, salt, and cold stresses.

However, 4 × M1.1 and 4 × M2.3 synthetic promoters were induced in response to mechanical wounding of leaves (Figure 7). During SCN and RKN infection process, infective juveniles enter host roots, puncture cells, and migrate throughout the root to initiate a feeding site. In our previous study, mechanical wounding of the transgenic hairy roots by multiple piercing with a needle did not result in induced expression of these synthetic promoters (Liu et al., 2014). Taken together, these results may suggest the systemic responses of plants to mechanical wounding of leaves by activating different sets of genes (Rojo et al., 1999). Particularly that no induction was observed by nematode-induced wounding in roots or in insect chewing leaves (Figures 2, 3; Supplementary Figure S6).

The SA and MeJA signaling pathways are prerequisites to the defense response against plant-pathogen interaction while ABA acts antagonistically and induces susceptibility to nematodes (Thaler and Bostock, 2004; López et al., 2008; De Torres Zabala et al., 2009; Robert-Seilaniantz et al., 2011; Nahar et al., 2012). Exogenous application of SA or MeJA induces root defense against RKN and SCN (Nahar et al., 2012). However, our study showed no inducibility of the synthetic promoters in response to SA treatment (Supplementary Figure S3). The sequence GGACTTTT is required for SA-responsive reporter gene expression (Lehmeyer et al., 2016), which is not contained in 4 × M1.1 and 4 × M2.3 synthetic promoters.

The 4 × M1.1 and 4 × M2.3 promoters exhibited increased GUS activity in leaves but not in roots after MeJA treatment (Supplementary Figure S4). The key enzymes (allene oxide synthase and allene oxide cyclase) for MeJA synthesis are initiated from the chloroplasts because these enzymes have chloroplast transit peptides (Rouster et al., 1997; Hu et al., 2009). Therefore, this might provide a possible explanation for induced GUS activity in leaves.

Furthermore, it has been shown that wounding plays important role in the induction of MeJA signal transduction pathway for defense response (Hu et al., 2009). This signaling molecule is responsible for wound or defense responses (Turner et al., 2002), and changes in expression of wound or defense response genes have been observed in several plant-nematode interactions (Gheysen and Fenoll, 2002). As such, the induced GUS activity in the leaves and roots by mechanical wounding might activated the defense signaling by MeJA, causing the responses to wounding and the subsequent upregulation of MeJA signaling.

Several studies reported that exogenous application of ABA leads to enhanced pathogen and nematode susceptibility (Koga et al., 2004; Jiang et al., 2010; Nahar et al., 2012). However, other studies showed the exogenous application of ABA promotes resistance in some pathogen and nematode interactions (Karimi et al., 1995; Asselbergh et al., 2008; Ton et al., 2009). It appears that ABA may be involved in nematode-infected roots and play important role in determining the effect of resistance or susceptibility. In our study, the ABA treatment resulted in increased GUS expression in the leaves under the control of 4 × M1.1 and 4 × M2.3 promoters after 24 h (Supplementary Figure S5). In contrast, no induced GUS activity was detected in the roots. ABA has been shown to play roles in cell division as well as in gene expression changes during host-nematode interaction (Phillips, 1971; Puzio et al., 2000; Mazarei et al., 2003).

The 4 × M1.1 and 4 × M2.3 promoters showed no induced GUS activity after insect (fall armyworm) treatment (Supplementary Figure S6). Although we observed the induction of synthetic promoters after mechanical wounding of leaves, this activation was not observed in insect-wounded tissues. These results suggest that the 4 × M1.1 and 4 × M2.3 promoters may not be sensitive to the damage by the insect. Several strong and rapid wound-inducible promoters (RbPCD1pro, pinIIpro, mpiC1pro) were used as an insect-inducible promoters (Godard et al., 2007; Tiwari et al., 2011; Pandey et al., 2019).

The sensitivity of the synthetic promoters was also examined in response to several bacterial pathogens. The inoculation of *P. syringae* pv. *glycinea* in soybean plants resulted in a slight increase in GUS expression in the leaves at 48 h compared to non-treated (Supplementary Figure S7). However, no induced GUS activity was observed in the roots (Supplementary Figure S7). The inoculation of soybean leaves with *P. syringae* pv. *glycinea* showed leaf spots with increased necrotic spots after 48 and 72 h of infection (Supplementary Figure S7). These observations further confirm *P. syringae* pv. *glycinea* as pathogen of soybean in a compatible interaction (Budde and Ullrich, 2000), causing slight inducibility in leaves but not in soybean roots after bacterial infection.

We observed no induction of the 4 × M1.1 and 4 × M2.3 promoters in response to *P. syringae* pv. *tomato* and *P. marginalis* at three different time points (Supplementary Figures S8, S9). Inoculation of soybean leaves with *P. syringae* pv. *tomato* led to a hypersensitive response (HR)-like response with rapid necrosis at 24 h and increased severity at later time points (Supplementary Figure S8; Budde and Ullrich, 2000; Collmer et al., 2000; Shan et al., 2000). These observations further confirm *P. syringae* pv. *tomato* as pathogenic bacteria in an incompatible interaction with soybean (Budde and Ullrich, 2000). No symptoms were observed after inoculation of soybean leaves with *P. marginalis* (Supplementary Figure S9), as expected for the bacteria causing the soft-rot diseases (Kůdela et al., 2011). Taken together, these results show that the synthetic promoters were not inducible in response to the non-host pathogens.

In conclusion, the present study has demonstrated that the 4 × M1.1 and 4 × M2.3 synthetic promoters are root-preferential directing GUS expression predominantly in the induced-nematode feeding structures. Additionally, the sensitivity of the promoters to nematode infection was shown to be specific as observed after exposure to a variety of biotic and abiotic stresses. These traits signify the potential use of these synthetic promoters in plant biotechnology and in developing nematode resistance in economically important crops.

## Data availability statement

The original contributions presented in the study are included in the article/Supplementary material, further inquiries can be directed to the corresponding author.

## Author contributions

MS designed and performed experiments, analyzed data, produced figures, and wrote the manuscript. MM participated in the experimental design, assisted with data analysis and interpretation, and wrote and revised the manuscript. RM assisted with the revisions and edits to the manuscript. WL developed synthetic promoter constructs. TH participated in the experimental design and assisted in conducting nematode experiments and interpreted the results. CS conceived and designed the study, acquired funding, and wrote and revised the manuscript. All authors contributed to the article and approved the submitted version.

## Funding

This research was funded by the Tennessee Soybean Promotion Board (TSPB) to CS, the USDA National Institute of Food and Agriculture Hatch project 02685 to WL, and Hatch projects to CS and TH.

## Conflict of interest

The authors declare that the research was conducted in the absence of any commercial or financial relationships that could be construed as a potential conflict of interest.

## Publisher’s note

All claims expressed in this article are solely those of the authors and do not necessarily represent those of their affiliated organizations, or those of the publisher, the editors and the reviewers. Any product that may be evaluated in this article, or claim that may be made by its manufacturer, is not guaranteed or endorsed by the publisher.
