# Supplementary material for: Functional analysis of soybean cyst nematode-inducible synthetic promoters and their regulation by biotic and abiotic stimuli in transgenic soybean (Glycine max)
Source: Front Plant Sci. 2022 Sep 9;13:988048. doi: 10.3389/fpls.2022.988048 (PMC9501883; doi:10.3389/fpls.2022.988048)
Supplement: Supplementary file 1 [file Data_Sheet_1.docx]

**Supplementary data**

**Functional analysis of soybean cyst nematode-inducible synthetic promoters and their regulation by biotic and abiotic stimuli in transgenic soybean (*Glycine max*)**

Mst Shamira Sultana^1,2^, Mitra Mazarei^1,2^, Reginald J. Millwood^1^, Wusheng Liu^3^, Tarek Hewezi^1^, C. Neal Stewart Jr.^1,2*^

^1^Department of Plant Sciences, University of Tennessee, Knoxville, Tennessee, USA

^2^Center for Agricultural Synthetic Biology, University of Tennessee, Knoxville, Tennessee, USA

^3^Department of Horticultural Science, North Carolina State University, Raleigh, North Carolina, USA

* Corresponding author: C. Neal Stewart Jr. Tel: 865-974-6487; Email: [nealstewart@utk.edu](mailto:nealstewart@utk.edu)

**Supplementary Table (S1):** Core motifs within the two selected synthetic promoters.

| **Promoter** | **Tetramerized motif sequences** | **Core motifs** |
| --- | --- | --- |
| 4×M1.1 | TAAAATAAAGTTCTTTAATT | TAAAA |
|  |  | TAAAGT |
|  |  | TCTTTA |
|  |  | TTAATT |
| 4×M2.3 | ATATAATTAAGT | ATATAA |
|  |  | TAATTA |
|  |  | ATTAAGT |
|  |  | AAGT |

**Supplementary Table (S2):** List of primers used in PCR and qRT-PCR.

| Gene | Forward primer | Reverse primer |
| --- | --- | --- |
| *Gus* | CGACTGGGCAGATGAACATG | GTTCAGGCACAGCACATCAA |
| *Bar* | GAAGTCCAGCTGCCAGAAAC | AAGCACGGTCAACTTCCGTA |
| *chvA* | CGAAACGCTGTTCGGCCTGTGG | GTTCAGCAGGCCGGCATCCTGG |
| *Gus* (qRT-PCR) | CTGCATCGGCGAACTGATC | GTCCTGTAGAAACCCCAACC |
| *GmUBI3* | GTGTAATGTTGGATGTGTTCCC | ACACAATTGAGTTCAACACAAACCG |


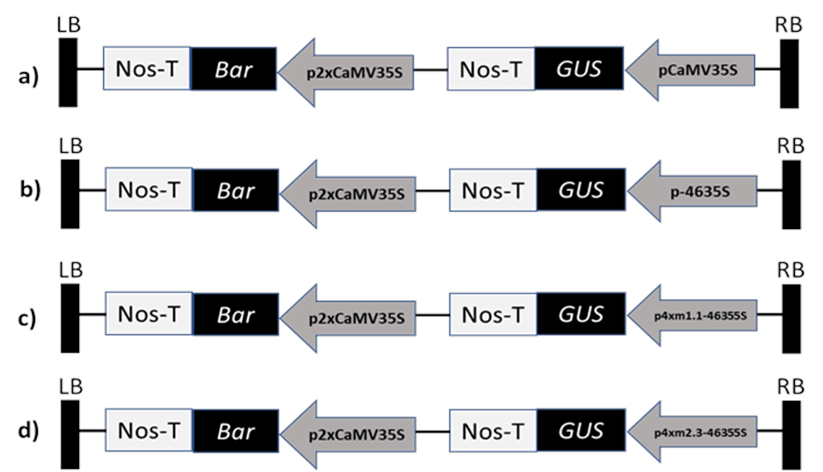


**Supplementary Figure (S1):** Schematic diagrams of promoter-GUS constructs. Left border (LB) and right border (RB) flanking the T-DNA insert are represented by black vertically elongated rectangles. 35SCaMV, 35S *Cauliflower mosaic virus* promoter; -4635S, minimal 35SCaMV promoter; 4×1.1 and 4×2.3, four repeats of the corresponding core motif; Nos-T, nopaline synthase terminator; Bar, bialaphos resistance gene; GUS, reporter gene.


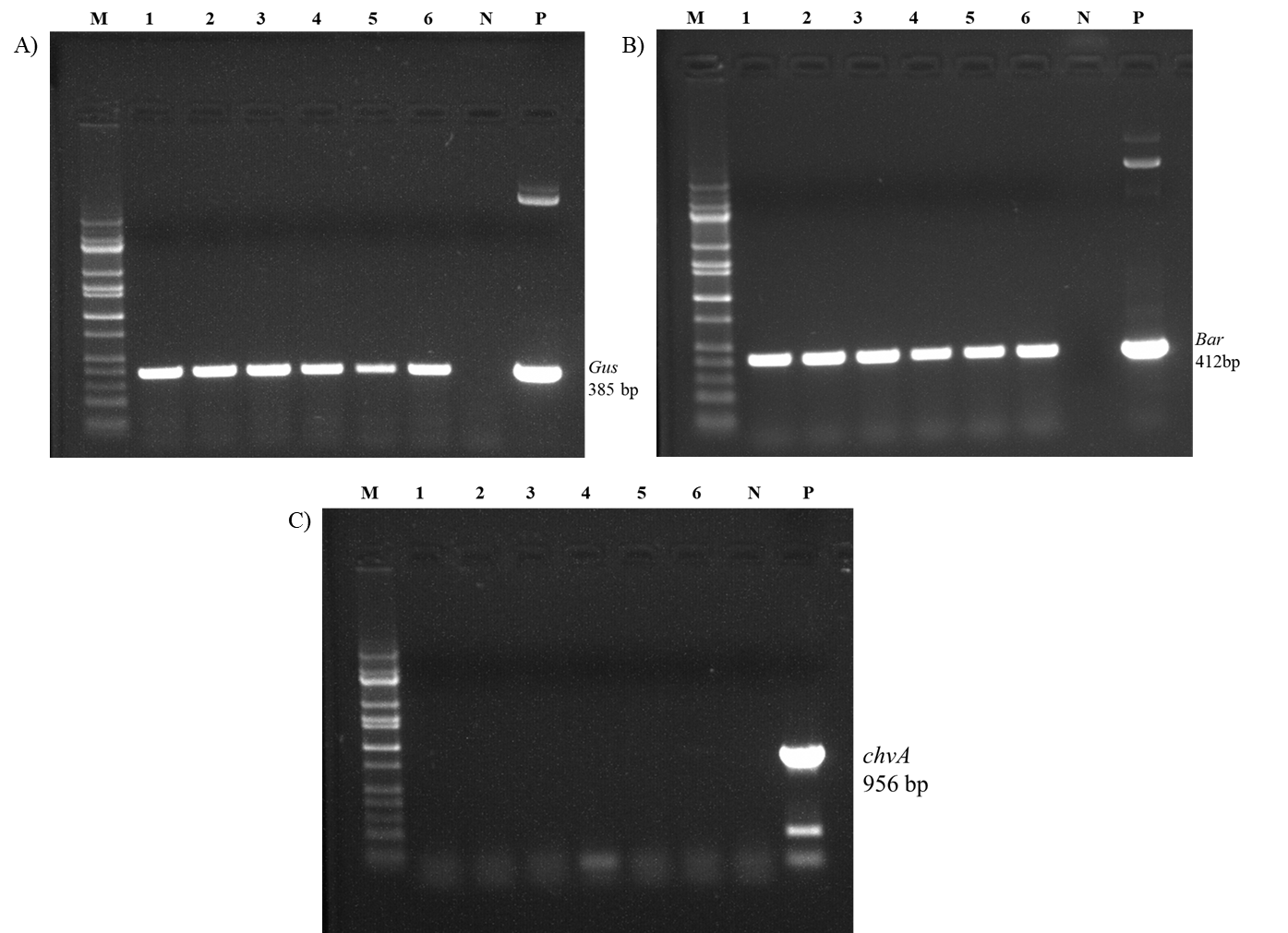


**Supplementary Figure (S2):** Molecular characterization of transgenic soybean plants containing promoter-GUS constructs. PCR analysis of genomic DNA extracted from leaves of 3-week-old plants. Lanes 1-3 are transgenic soybean lines L1, L2, and L3 containing 4×M1.1 promoter construct. Lanes 4-6 are transgenic soybean lines L1, L2, and L3 containing 4×M2.3 promoter construct. Lane N is non-transgenic wild-type soybean. Lane P is positive control template vector plasmid. The expected amplified DNA band size indicate presence of **(A)** GUS gene (385 bp) and **(B)** Bar gene (412 bp). *chvA* gene **(C)** was used as a control for the *Agrobacterium* contamination. No amplification was observed in transgenic lines **(C)**. M, DNA marker.


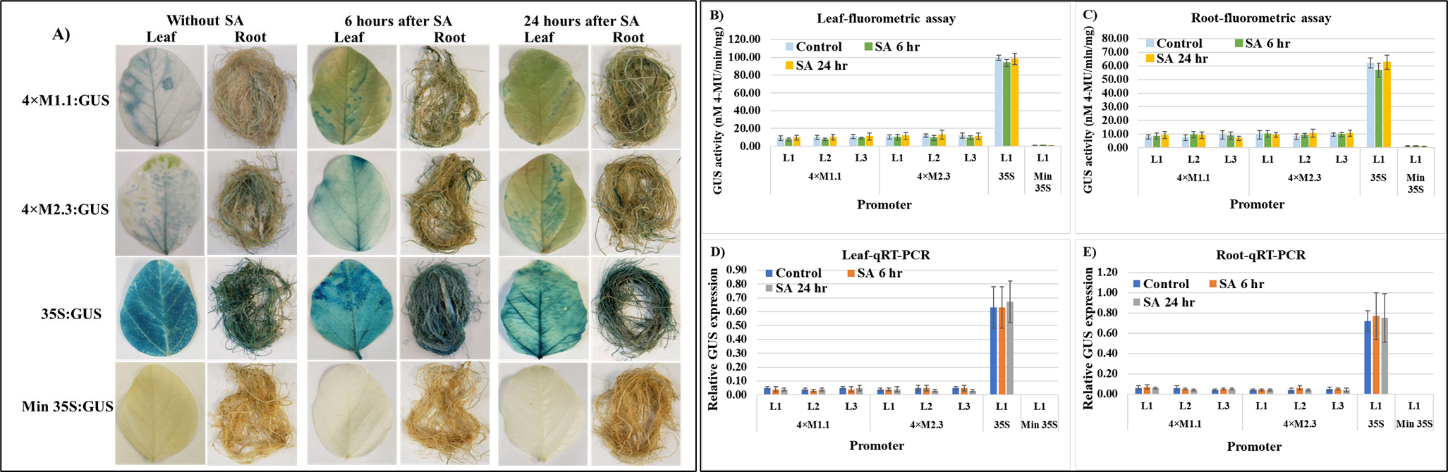


**Supplementary Figure (S3):** GUS activity in leaves and roots of 3-week-old T_3_ transgenic soybean containing the individual promoter-GUS construct subjected to mock (untreated control) and salicylic acid (SA) (100 µM) at two time points of 6 h and 24 h after treatment. **(A)** Histochemical staining for GUS activity in transgenic soybean plants. **(B, C)** Fluorometric assay for GUS activity in leaf **(B)** and root **(C)**. **(D, E)** Quantitative real-time RT-PCR (qRT-PCR) analysis for GUS expression in leaf **(D)** and root **(E)**. The relative levels of transcripts were normalized to soybean ubiquitin gene (*GmUBI*3). Three independent transgenic lines (L1, L2, and L3) were used for 4×M1.1 and 4×M2.3 promoter-GUS constructs. One transgenic line (L1) was used for 35S and minimal (Min) 35S promoter-GUS constructs. Bars represent mean values of six biological replicates (plants) ± standard error. Statistical analysis by a two-sample paired t-test (*P* <0.05) indicated no significant differences between treated and untreated plants.


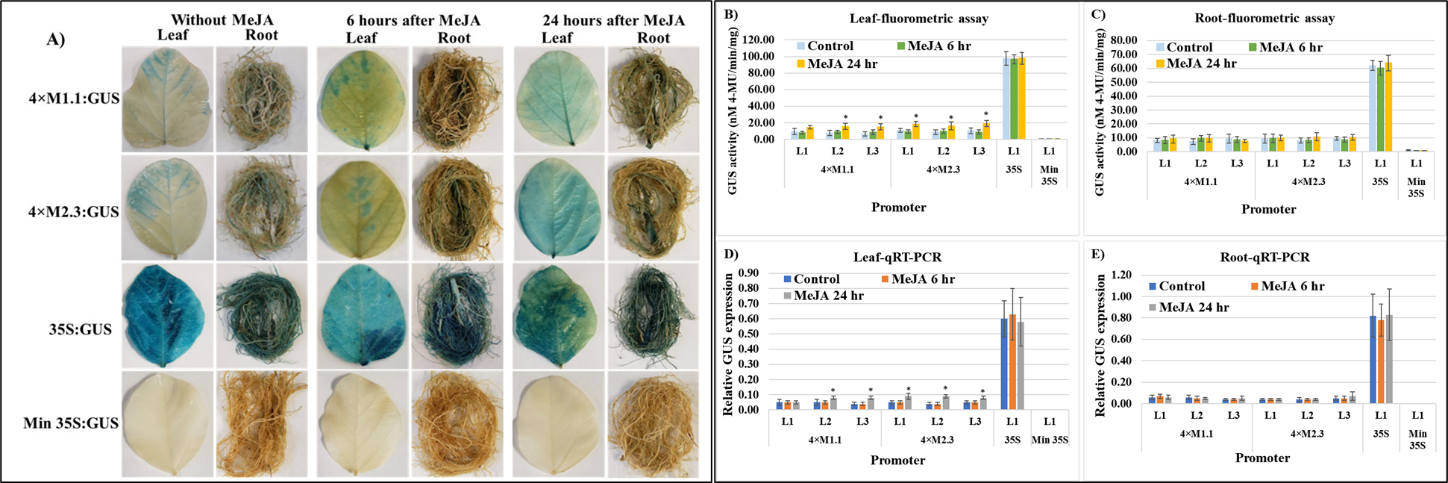


**Supplementary Figure (S4):** GUS activity in leaves and roots of 3-week-old T_3_ transgenic soybean containing the individual promoter-GUS construct subjected to mock (untreated control) and methyl jasmonate (MeJA) (100 µM) at two time points of 6 h and 24 h after treatment. **(A)** Histochemical staining for GUS activity in transgenic soybean plants. **(B, C)** Fluorometric assay for GUS activity in leaf **(B)** and root **(C)**. **(D, E)** Quantitative real-time RT-PCR (qRT-PCR) analysis for GUS expression in leaf **(D)** and root **(E)**. The relative levels of transcripts were normalized to soybean ubiquitin gene (*GmUBI*3). Three independent transgenic lines (L1, L2, and L3) were used for 4×M1.1 and 4×M2.3 promoter-GUS constructs. One transgenic line (L1) was used for 35S and minimal (Min) 35S promoter-GUS constructs. Bars represent mean values of six biological replicates (plants) ± standard error. Statistical significance (*p* < 0.05) was determined by two-sample paired t-test. Bars with asterisk (*) indicate significant difference compared to untreated control plants.


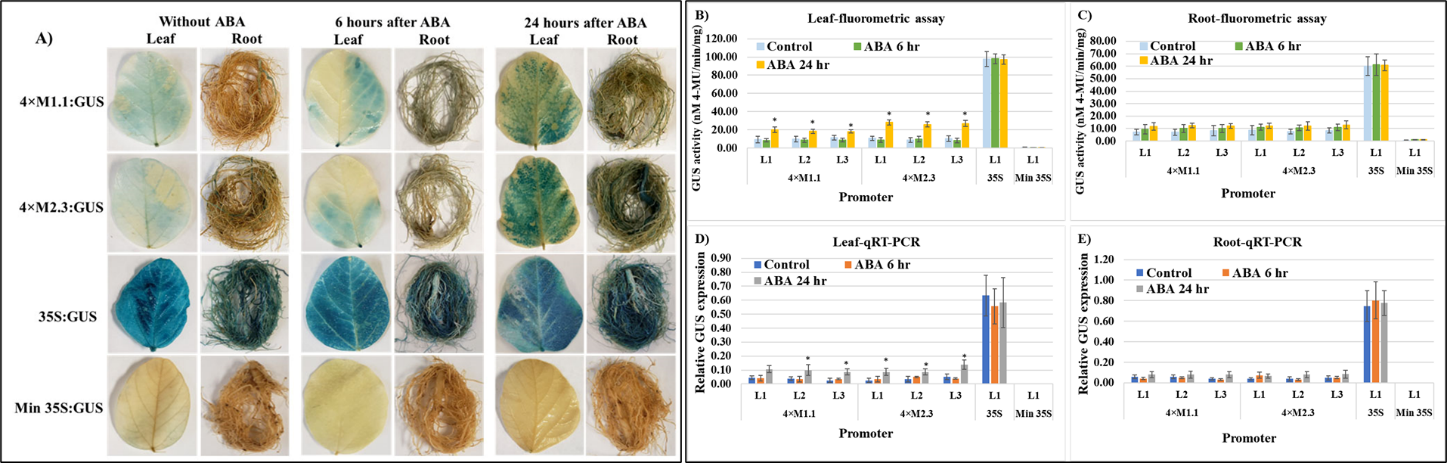


**Supplementary Figure (S5).** GUS activity in leaves and roots of 3-week-old T_3_ transgenic soybean containing the individual promoter-GUS construct subjected to mock (untreated control) and abscisic acid (ABA) (100 µM) at two time points of 6 h and 24 h after treatment. **(A)** Histochemical staining for GUS activity in transgenic soybean plants. **(B, C)** Fluorometric assay for GUS activity in leaf **(B)** and root **(C)**. **(D, E)** Quantitative real-time RT-PCR (qRT-PCR) analysis for GUS expression in leaf **(D)** and root **(E).** The relative levels of transcripts were normalized to soybean ubiquitin gene (*GmUBI*3). Three independent transgenic lines (L1, L2, and L3) were used for 4×M1.1 and 4×M2.3 promoter-GUS constructs. One transgenic line (L1) was used for 35S and minimal (Min) 35S promoter-GUS constructs. Bars represent mean values of six biological replicates (plants) ± standard error. Statistical significance (*p* < 0.05) was determined by two-sample paired t-test. Bars with asterisk (*) indicate significant difference compared to untreated control plants.


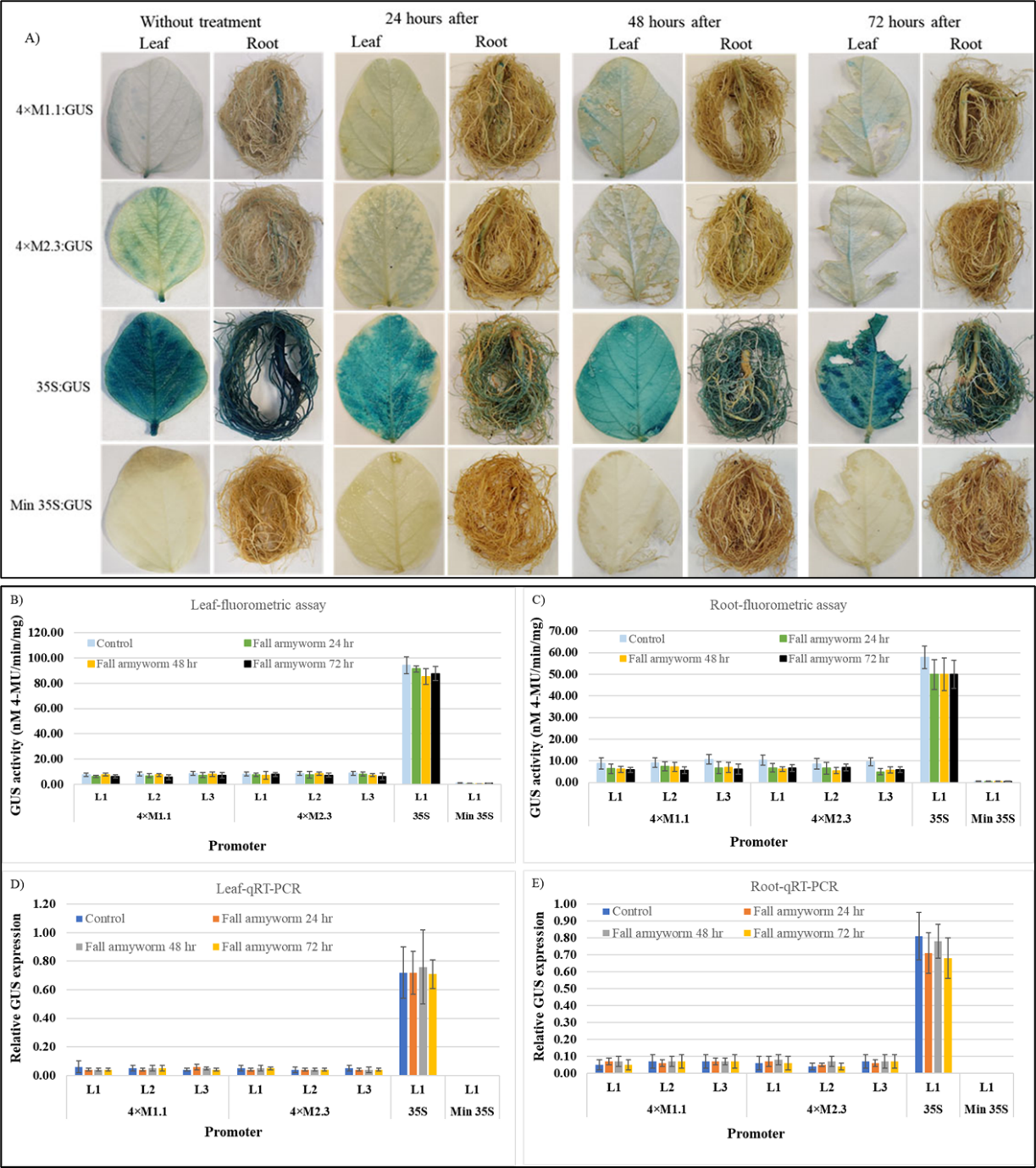


**Supplementary Figure (S6):** GUS activity in leaves and roots of transgenic soybean containing the individual promoter-GUS construct untreated or treated with fall armyworm (*Spodoptera frugiperda*) larvae at 24, 48, and 72 h after larval infestation. **(A)** Histochemical staining for GUS activity in transgenic soybean plants. **(B, C)** Fluorometric assay for GUS activity in leaf **(B)** and root **(C).** **(D, E)** Quantitative real-time RT-PCR (qRT-PCR) analysis for GUS expression in leaf **(D)** and root **(E)**. The relative levels of transcripts were normalized to soybean ubiquitin gene (*GmUBI*3). Three independent transgenic lines (L1, L2, and L3) were used for 4×M1.1 and 4×M2.3 promoter-GUS constructs. One transgenic line (L1) was used for 35S and minimal (Min) 35S promoter-GUS constructs. Bars represent mean values of six biological replicates (plants) ± standard error. Statistical analysis by a two-sample paired t-test (*P* <0.05) indicated no significant differences between treated and untreated plants.


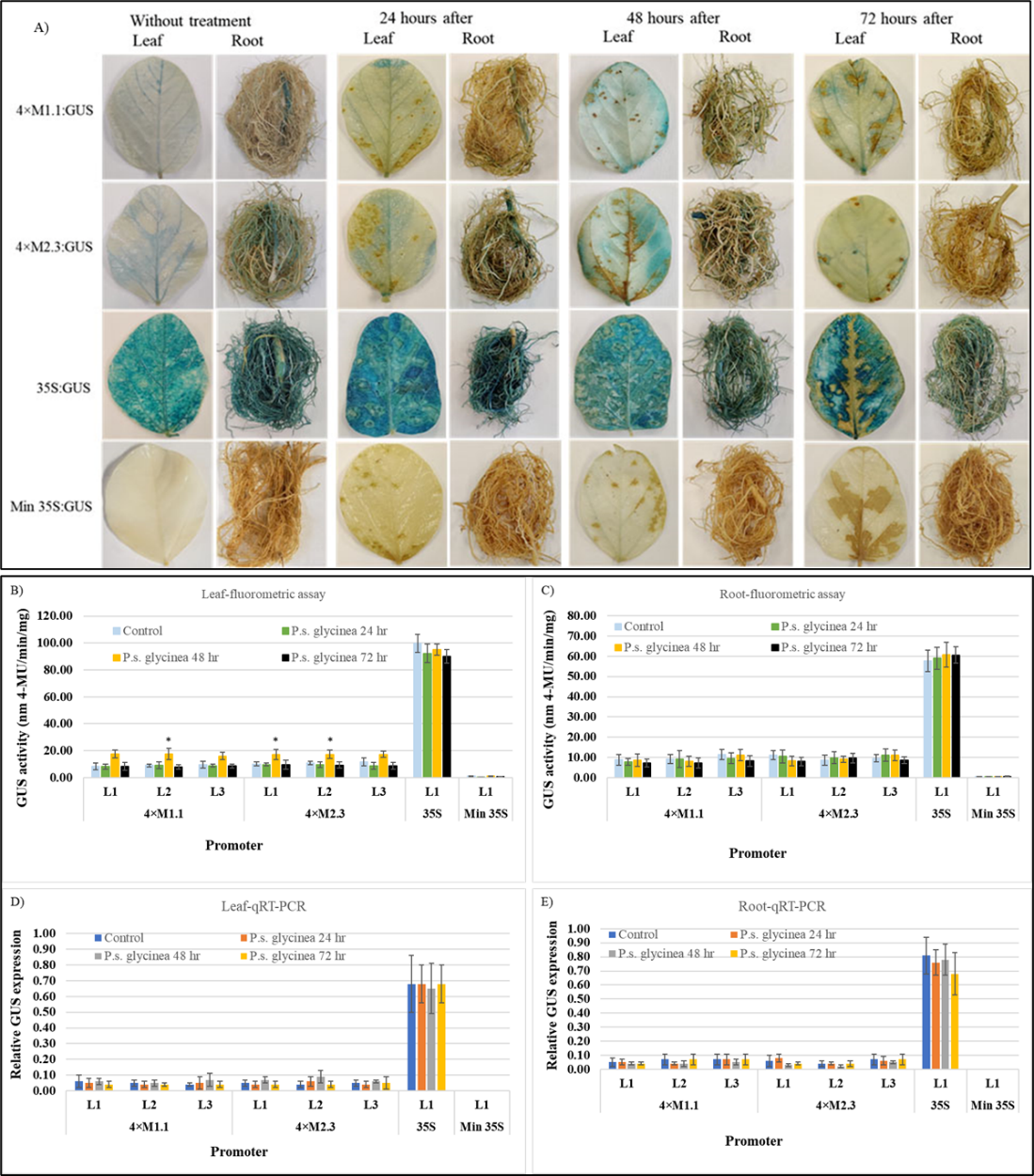


**Supplementary Figure (S7):** GUS activity in leaves and roots of transgenic soybean containing the individual promoter-GUS construct treated with 10 mM MgCl_2_ (mock control) or *pseudomonas syringe* pv. *glycinea* at 24, 48, and 72 h after treatment. **(A)** Histochemical staining for GUS activity in transgenic soybean plants. **(B, C)** Fluorometric assay for GUS activity in leaf **(B)** and root **(C)**. **(D, E)** Quantitative real-time RT-PCR (qRT-PCR) analysis for GUS expression in leaf **(D)** and root **(E)**. The relative levels of transcripts were normalized to soybean ubiquitin gene (*GmUBI*3). Three independent transgenic lines (L1, L2, and L3) were used for 4×M1.1 and 4×M2.3 promoter-GUS constructs. One transgenic line (L1) was used for 35S and minimal (Min) 35S promoter-GUS constructs. Bars represent mean values of six biological replicates (plants) ± standard error. Statistical significance (*p* < 0.05) was determined by two-sample paired t-test. Bars with asterisk (*) indicate significant difference compared to untreated control plants.


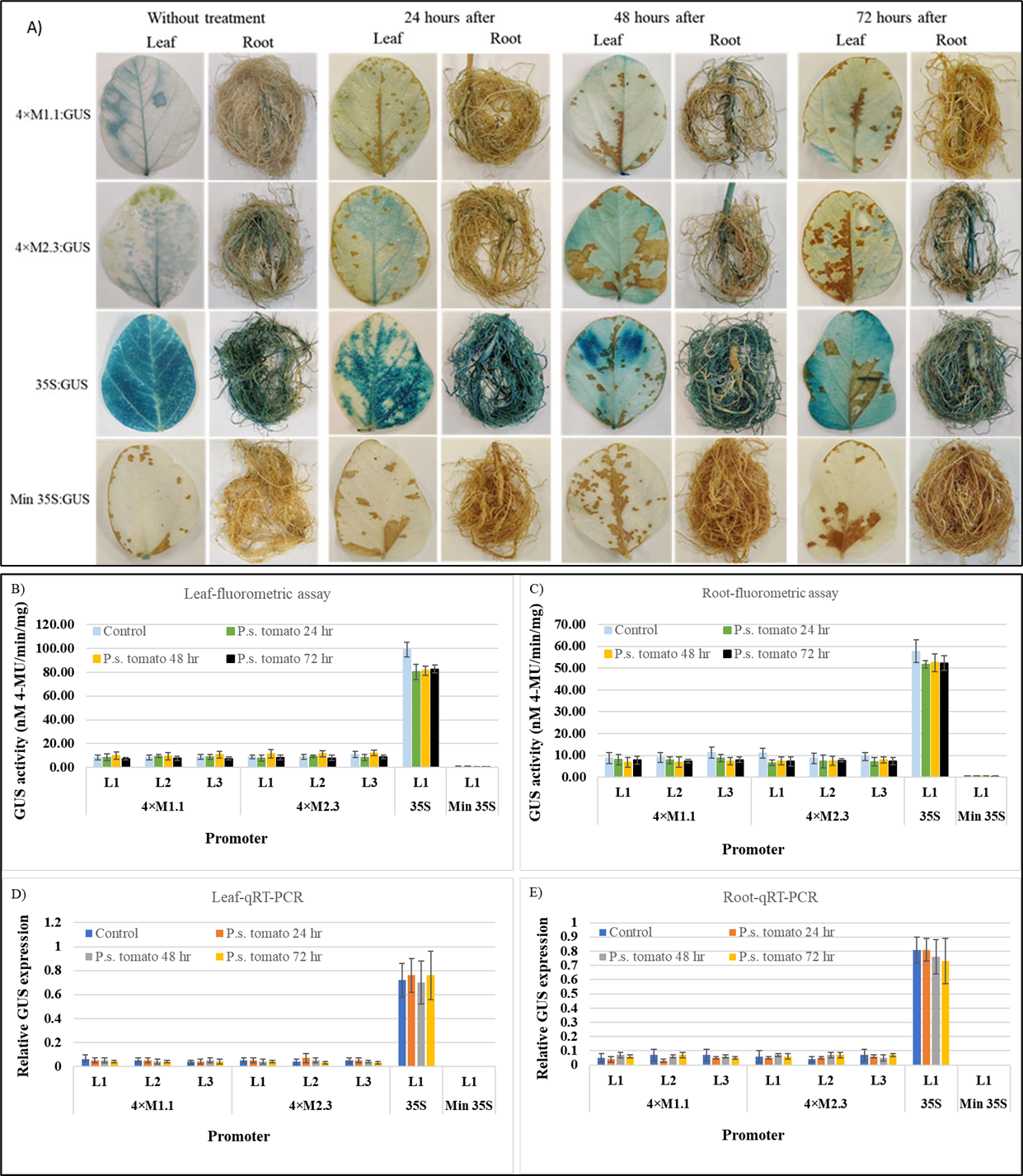


**Supplementary Figure (S8):** GUS activity in leaves and roots of transgenic soybean containing the individual promoter-GUS construct treated with 10 mM MgCl_2_ (mock control) or *Pseudomonas syringe* pv. *tomato* at 24, 48, and 72 h after treatment. **(A)** Histochemical staining for GUS activity in transgenic soybean plants. **(B, C)** Fluorometric assay for GUS activity in leaf **(B)** and root **(C)**. **(D, E)** Quantitative real-time RT-PCR (qRT-PCR) analysis for GUS expression in leaf **(D)** and root **(E)**. The relative levels of transcripts were normalized to soybean ubiquitin gene (*GmUBI*3). Three independent transgenic lines (L1, L2, and L3) were used for 4×M1.1 and 4×M2.3 promoter-GUS constructs. One transgenic line (L1) was used for 35S and minimal (Min) 35S promoter-GUS constructs. Bars represent mean values of six biological replicates (plants) ± standard error. Statistical analysis by a two-sample paired t-test (*P* <0.05) indicated no significant differences between treated and untreated plants.


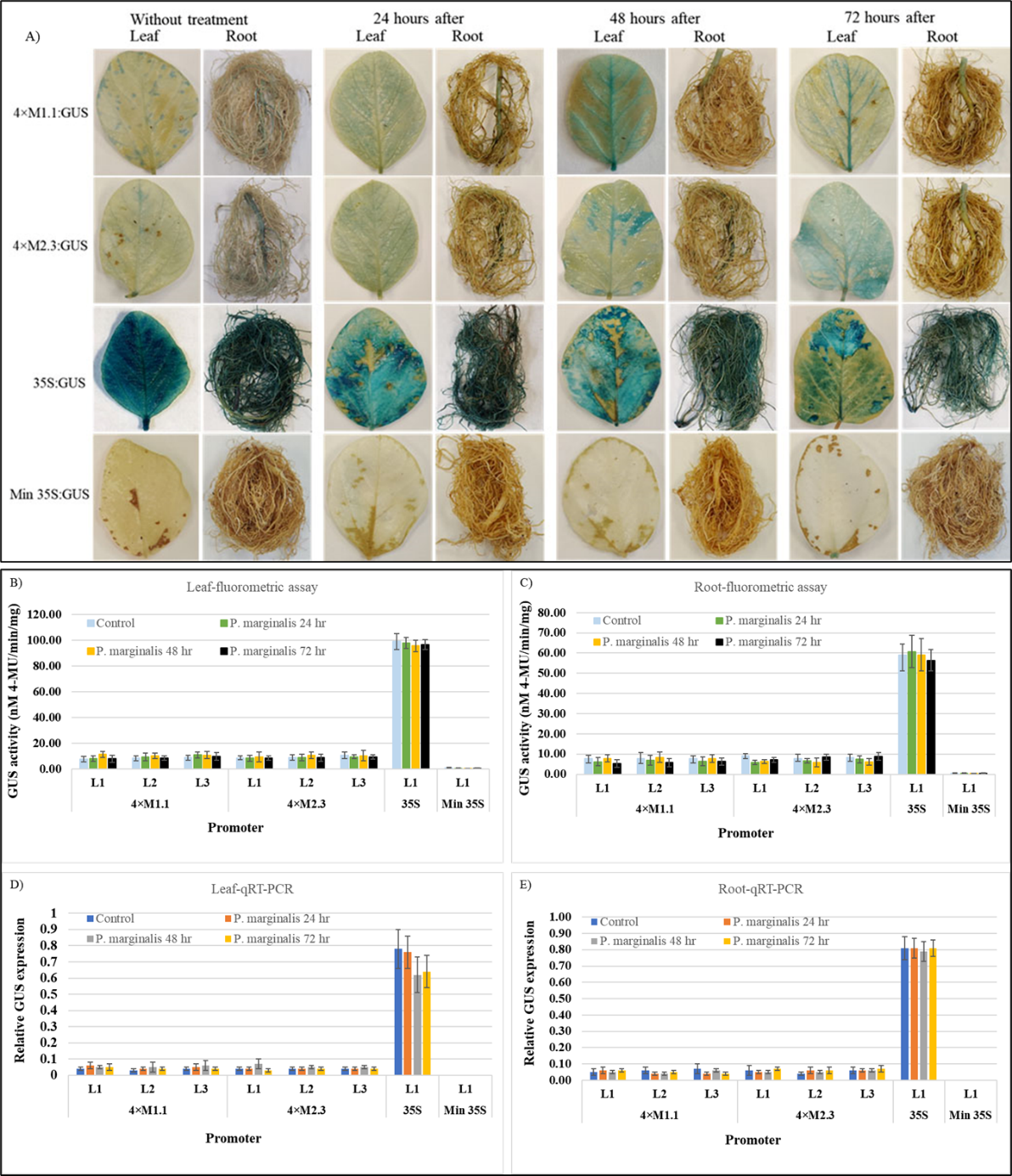


**Supplementary Figure (S9):** GUS activity in leaves and roots of transgenic soybean containing the individual promoter-GUS construct treated with 10 mM MgCl_2_ (mock control) or *Pseudomonas marginalis* at 24, 48, and 72 h after treatment. **(A)** Histochemical staining for GUS activity in transgenic soybean plants. **(B, C)** Fluorometric assay for GUS activity in leaf **(B)** and root **(C)**. **(D, E)** Quantitative real-time RT-PCR (qRT-PCR) analysis for GUS expression in leaf **(D)** and root **(E)**. The relative levels of transcripts were normalized to soybean ubiquitin gene (*GmUBI*3). Three independent transgenic lines (L1, L2, and L3) were used for 4×M1.1 and 4×M2.3 promoter-GUS constructs. One transgenic line (L1) was used for 35S and minimal (Min) 35S promoter-GUS constructs. Bars represent mean values of six biological replicates (plants) ± standard error. Statistical analysis by a two-sample paired t-test (*P* <0.05) indicated no significant differences between treated and untreated plants.
